# Emodin Protects Against Lipopolysaccharide-Induced Acute Lung Injury *via* the JNK/Nur77/c-Jun Signaling Pathway

**DOI:** 10.3389/fphar.2022.717271

**Published:** 2022-03-17

**Authors:** Pei Xie, Li-Jun Yan, Hong-Ling Zhou, Hui-Hui Cao, Yuan-Ru Zheng, Zi-Bin Lu, Hua-Yi Yang, Jia-Mei Ma, Yu-Yao Chen, Chuying Huo, Chunyang Tian, Jun-Shan Liu, Lin-Zhong Yu

**Affiliations:** ^1^ Third Level Research Laboratory of State Administration of Traditional Chinese Medicine, School of Traditional Chinese Medicine, Southern Medical University, Guangzhou, China; ^2^ Guangdong Provincial Key Laboratory of Chinese Medicine Pharmaceutics, Guangzhou, China

**Keywords:** acute lung injury, inflammation, lipopolysaccharide, emodin, JNK/Nur77/c-Jun

## Abstract

**Background:** Acute lung injury (ALI) is a serious inflammatory disease with clinical manifestations of hypoxemia and respiratory failure. Presently, there is no effective treatment of ALI. Although emodin from *Rheum palmatum* L. exerts anti-ALI properties, the underlying mechanisms have not been fully explored.

**Purpose:** This study aimed to investigate the therapeutic effect and mechanism of emodin on LPS-induced ALI in mice.

**Methods:** RAW264.7 cells and zebrafish larvae were stimulated by LPS to establish inflammatory models. The anti-inflammatory effect of emodin was assessed by ELISA, flow cytometric analysis, and survival analysis. *In vitro* mechanisms were explored by using Western blotting, luciferase assay, electrophoretic mobility shift assay (EMSA), and small interfering RNA (siRNA) approach. The acute lung injury model in mice was established by the intratracheal administration of LPS, and the underlying mechanisms were assessed by detecting changes in histopathological and inflammatory markers and Western blotting in lung tissues.

**Results:** Emodin inhibited the inflammatory factor production and oxidative stress in RAW264.7 cells, and prolonged the survival of zebrafish larvae after LPS stimulation. Emodin suppressed the expression levels of phosphorylated JNK at Thr183/tyr182 and phosphorylated Nur77 at Ser351 and c-Jun, and increased the expression level of Nur77 in LPS-stimulated RAW264.7 cells, while these regulatory effects of emodin on Nur77/c-Jun were counteracted by JNK activators. The overexpression of JNK dampened the emodin-mediated increase in Nur77 luciferase activity and Nur77 expression. Moreover, the inhibitory effect of emodin on c-Jun can be attenuated by Nur77 siRNA. Furthermore, emodin alleviated LPS-induced ALI in mice through the regulation of the JNK/Nur77/c-Jun pathway.

**Conclusions:** Emodin protects against LPS-induced ALI through regulation on JNK/Nur77/c-Jun signaling. Our results indicate the potential of emodin in the treatment of ALI.

## Introduction

ALI is a deadly inflammatory disease that is mainly characterized by excessive neutrophil infiltration and pro-inflammatory cytokines release, which further causes endothelial and epithelial barrier disruption (Rubenfeld et al., 2005; Fanelli and Ranieri, 2015). The existing therapeutic strategies for ALI are respiratory support and anti-inflammatory therapies, including the inhibition of the release of inflammatory cytokines and the protection of the endothelial and epithelial barrier from excessive inflammation-induced damage (Johnson and Matthay, 2010). Despite the improvement in the diagnosis and treatment of ALI in the past few years, the mortality of ALI remains high (Lalu et al., 2014). Thus, it is urgent to explore novel therapy strategies for ALI.

Multiple signaling pathways are involved in inflammatory responses, including MAPK, STAT3, and Nur77/c-Jun. The MAPK pathway consists of a well-studied family of serine/threonine protein kinases that transmit extracellular stimuli into intracellular ROS by phosphorylation (Ha et al., 2018). There are three subfamilies of MAPKs in mammals: ERK, p38, and JNK. Among them, JNK is closely involved in inflammatory responses (Mehan et al., 2011). It is reported that JNK inhibition protected against the ALI animal models by ameliorating lung histopathologic changes and lung edema, reducing inflammatory cell infiltration, and inhibiting the secretion of pro-inflammatory cytokines TNF-α and interleukin (IL-6) (Shen et al., 2017; Jin and Jin, 2018).

Previous studies showed that LPS-induced JNK activation can regulate Nur77 and then promote inflammation (Hongling Zhou et al., 2019). Nur77 (NR4A1), also known as the nerve growth factor-induced gene B or TR3, is a member of the NR4A subfamily that belongs to orphan nuclear receptors (To et al., 2012). Nur77 is involved in a variety of cellular processes, including proliferation, differentiation, apoptosis, metabolism, and inflammation (Pearen and Muscat, 2010). Nur77 can be induced as an early response gene by inflammatory stimuli, including LPS, cytokines, or oxidized lipids in macrophages (Pei et al., 2005; Maxwell and Muscat, 2006). The deficiency of Nur77 induces macrophage differentiation toward the M1 pro-inflammatory phenotype (Hanna et al., 2012). In an *in vivo* study, the activation of Nur77 alleviated LPS-induced ALI due to the inhibition of endothelin-1 (Jiang et al., 2016). However, the role of Nur77 in ALI is unclear.

Traditional Chinese medicine *Rheum palmatum* L. (Rhubarb) has been widely used for treating inflammatory diseases for thousands of years in China. Previous research reported that *Rheum palmatum* L. combined with routine comprehensive treatment remarkedly decreased the mortality of patients with acute respiratory distress syndrome by reducing the mechanical ventilation time, ameliorating the arterial blood gas and the cytokine levels (Yang et al., 2017). Emodin, a main effective ingredient of *Rhizoma palmatum* L., has been reported to protect against ALI animal models, which is associated with the downregulation of the pre-B-cell colony-enhancing factor, upregulation of aquaporin 1 and aquaporin 2, and inhibition of NF-κB (Xiao et al., 2014; Xu et al., 2016; Cui et al., 2017).

In this study, we first demonstrate that emodin possesses anti-inflammatory activities *in vivo* and *in vitro* by inhibiting the JNK pathway, and subsequently regulating Nur77/c-Jun signaling. These findings indicate that emodin has the potential to be developed as a novel drug to treat ALI and provide strong evidence for the crucial role of the JNK/Nur77/c-Jun pathway in inflammation.

## Materials and Methods

### Materials

Emodin was purchased from Aladdin (Shanghai, China). Dexamethasone was obtained from Tianxin Pharmaceutical (Guangzhou, China). The RPMI-1640 medium, Dulbecco- modified Eagle medium, fetal bovine serum, penicillin–streptomycin, BCA Protein Assay Kit, MTT, LightShift™ Chemiluminescent EMSA Kit, enhanced chemiluminescence (ECL) kit, and the Opti-MEN medium were obtained from Thermo Fisher Scientific (Waltham, USA). SP600125 and anisomycin were purchased from Selleckchem (Houston, USA). The Lipofectamine^®^ 2000 transfection reagent was purchased from Invitrogen (Grand Island, USA). Antibodies against p38, phosphor p38 (Thr180/Tyr182), JNK, *p*-JNK (Thr183/Tyr185), ERK, phosphor ERK (Thr202/Tyr204), phosphor Nur77 (Ser351), c-Jun, and phosphor c-Jun (Ser73) were purchased from Cell Signaling Technology (Danvers, USA). The antibody against Nur77 was purchased from Abcam (Cambridge, UK). Anti-β-actin was obtained from Boster (Wuhan, China). LPS and other reagents were obtained from Sigma-Aldrich (St. Louis, USA). JNK1 plasmid, mouse Mapk8 (NCBI Reference Sequence: NM_001310453.1) with a vector of pcDNA3.1 (+), was purchased from GenePharma (Shanghai, China).

### Cell Culture

The murine macrophage cell line RAW264.7 and human renal epithelial cell line HEK293T were obtained from American Type Culture Collection (ATCC, Rockville, USA), and were maintained at a humidified incubator containing 5% CO_2_ (*v/v*) at 37°C in RPMI-1640 or DMEM (Thermo Fisher Scientific, Massachusetts, USA), respectively, supplemented with 10% (*v/v*) fetal bovine serum (Thermo Fisher Scientific, Massachusetts, USA) and 1.0% (*v/v*) penicillin–streptomycin (Thermo Fisher Scientific, Massachusetts, USA).

### Cell Viability Assay

The MTT assay was used to measure the cell viability as previously described (Liu et al., 2016). Briefly, RAW264.7 cells were seeded in 96-well culture plates at a density of 6×10^3^ cells per well for 24 h and then treated with emodin (0, 10, 20, 30, 40, or 50 μM) for 24 h. Then, 30 μL of 3-[4,5-dimethylthiazol-2-yl]-2,5-diphenyltetrazoliumbromide (MTT) solution (5 mg/ml in PBS) was added to each well, and cells were incubated for another 4 h. Next, the supernatant was discarded, and 100 μL of DMSO was added to dissolve the formazan crystal. The OD values were measured by using an absorbance microplate reader (Thermo Fisher Scientific, Massachusetts, USA) at a wavelength of 570 nm.

### Enzyme-Linked Immunosorbent Assay

RAW264.7 cells were seeded in 24-well plates at a density of 1 × 10^5^ per well and cultured overnight. Then the cells were pretreated with indicated concentrations of emodin (0, 30, 35, or 40 μM) for 2 h before being stimulated with or without LPS (100 ng/ml) for another 18 h. The concentrations of TNF-α, IL-6 (Dakewei, Shang hai, China), MCP-1, and MIP-2 in the supernatant were detected by using ELISA kits. The absorbance was measured at 450 nm by using an absorbance microplate reader (Thermo Fisher Scientific, Wastham, USA).

### Flow Cytometric Analysis

RAW264.7 cells were seeded in 24-well plates at a density of 1 × 10^5^ per well and cultured overnight. Then the cells were pretreated with indicated concentrations of emodin (0, 30, 35, or 40 μM) for 2 h before being stimulated with or without LPS (100 ng/ml) for another 18 h. DCFH-DA does not fluoresce itself but can pass through the cell membrane freely. After entering the cell, DCFH can be hydrolyzed by the esterase in the cell to generate DCFH. Intracellular reactive oxygen species can oxidize non-fluorescent DCFH to produce fluorescent DCF. The treated RAW264.7 cells were raised with 3.3 μM DCFH–DA (S0033, Beyotime) in a serum-free medium for 20 min at 37°C and washed three times with PBS. The level of ROS was estimated as the percent of DCF fluorescence according to the negative control, which was determined by flow cytometry (CytoFLEX, Beckman Coulter, USA). To evaluate the level of intracellular ROS, each group was compared with the negative group. According to the size of FSC, the larger cells of FSC were set as the P1 region to remove cell fragments. In the figure of fluorescence and FITC, the boundary between positive and negative was delineated according to the fluorescence intensity of the negative group. The positive area was denoted as P2, and the ratio of P2 to P1 was the ROS level of each group.

### Zebrafish Maintenance

The zebrafish line *Tg (mpo:GFP)* was provided by the Key Laboratory of Zebrafish Modeling and Drug Screening for Human Diseases Institute at Southern Medical University (Guangzhou, China). Adult zebrafish were maintained in a recirculating tank rack system with a 14 h light/10 h dark cycle at 28°C in a certificated zebrafish facility (PENTAIR, USA) (Junyi Zhou et al., 2019). The larvae were maintained with a 14 h light/10 h dark cycle at 28.5°C in an incubator (Zhicheng, Shanghai, China).

### LPS-Induced Acute Inflammation in Zebrafish Larvae

At 3 days post-fertilization (dpf), the zebrafish larvae were collected and anesthetized with 0.02% tricaine, and subsequently microinjected with 2 nL of LPS (0.5 mg/ml) into the yolk to induce an acute inflammatory response (Yang et al., 2014). 1 h post-injection (hpi), the larvae were moved to a 6-well plate with emodin (0.05 μM) or dexamethasone (DEX, 5 μg/ml) in egg water. The larvae that were yolk-microinjected with PBS were used as the negative control. Next, the mortality of the zebrafish was recorded within 72 hpi.

### Western Blotting

After treatment, the cells were harvested and lysed with a lysis buffer (50 mM Tris, pH 7.5, 150 mM NaCl, 1% Triton X-100, 1 mM EDTA, 1 mM PMSF, 1 mM Na_3_VO_4_, 1 mM dithiothreitol, and 1 mM phosphatase inhibitor) for 15 min at 4°C, and then the lysates were centrifuged at 15,000 ×*g* for 20 min at 4°C, and the supernatant was collected as total cell lysates. For nuclear protein extraction, the cells were lysed with the nucleoprotein extraction kit (Sangon, shanghai, China) as described previously (Liu et al., 2016). Protein concentrations were measured by the BCA Assay Kit (Thermo Fisher Scientific, Massachusetts, USA). Proteins were separated by 10% SDS polyacrylamide gel electrophoresis (PAGE) and transferred onto PVDF membranes (Bio-Rad, Hercules, USA). After non-specific blocking with 5% skin milk (BD, New Jersey, USA) dissolved in Tris-buffered saline containing 0.05% (*v/v*) Tween-20 (TBS-T) for 1 h at room temperature (RT), the membranes were incubated with primary antibodies diluted in TBS-T containing 5% (*v/v*) BSA at 4°C overnight. Subsequently, membranes were washed with TBS-T three times and incubated with appropriate secondary antibodies (1:1000) for 1 h at RT (Lu et al., 2018). Immunoreactive bands were detected by an ECL kit by FluorChem^E^ (ProteinSimple, San Francisco, USA).

### Small Interfering RNA Transient Transfection

RAW264.7 cells were cultured in 6-well plates at a density of 4 × 10^5^ cells per well for 24 h and then transfected with a siRNA duplexe murine Nur77 (120 nM) or negative siRNA (120 nM) by Lipofectamine^®^ 2000, according to the manufacturer’s instruction. After that, the cells were treated with 40 μM emodin for 2 h before being stimulated with LPS (100 ng/ml) for another 18 h. The transfection efficiency was detected by fluorescein-labeled non-targeted siRNA.

### Electrophoretic Mobility Shift Assay

The DNA-binding activity of c-Jun was assayed by EMSA according to the manufacturer’s introduction. The c-Jun sequence of biotin-labeled and unlabeled double-stranded oligonucleotide probe was 5′-GG GCT TGA TGA GTC AGC CGG ACC-3'. Nuclear proteins (20 μg) were incubated with 6 μL of DNA-binding reaction buffer (ThermoFisher Scientific, Massachusetts, USA) in the presence of a c-Jun biotin-labeled probe and (or) unlabeled double probe (Genepharma, shanghai, China) for 20 min at RT. Then, the mixture was separated by 6.5% on-denaturing PAGE and then transferred onto a nylon membrane (Merck, Darmstadt, Germany). Subsequently, the membrane was exposed to a UVP Crosslinker (Analytic Jena, Jena, Germany) at 12,000 J cm^−2^ twice for DNA-protein crosslinking. The membrane was incubated with a blocking buffer for 15 min before being incubated with a blocking buffer containing horseradish peroxidase-conjugated streptavidin (1:300) for another 15 min. Having been washed with 1×washing buffer for 5 min, the DNA–protein complex on the membrane was visualized by using an ECL kit by FluorChem^E^ (ProteinSimple, San Francisco, USA).

### Plasmid Transfection and Luciferase Assay

HEK293.7T cells were co-transfected with Nur77 luciferase reporter plasmid (Gene Pharma, Shanghai, China) and *Renilla* luciferase reporter plasmid (GenePharma, Shanghai, China), in the presence or absence of JNK expression plasmid for 16 h using a Lipofectamine^®^ 2000 reagent (Invitrogen, Carlsbad, USA). 16 h after transfection, cells were treated with emodin (40 μM) for another 8 h. After being lysed with 100 μL of 1 × passive lysis buffer (Promega, Madison, USA) for 15 min, cells lysates (20 μL) were subjected to a luciferase assay by using a dual-luciferase reporter assay system (Promega, Madison, USA) according to the manufacturer’s instruction. The luciferase activity was normalized for transfection efficiency by the corresponding *Renilla* luciferase activity.

### Mouse

5- to 6-week-old male BALB/c mice (18–22 g) were obtained from the Experimental Animals Center of Southern Medical University (Guangzhou, China). The mice were housed in climate-controlled quarters (22–26°C at 40–70% humidity) and a 12 h light/12 h dark cycle with free food and water. All animal care and experimental protocols were approved by the Ethical Committee of Southern Medical University (No. L2018246). All studies involving animals were in compliance with the National Institutes of Health Guide for the Care and Use of Laboratory Animals.

### Establishment of Acute Lung Injury Models in BALB/c Mice

48 male BALB/c mice (18–22 g) were randomly allocated into 6 groups (*n* = 8 per group):

Control group: Mice were intragastrically administrated with 200 μL of saline once a day for 7 consecutive days. Then, they were treated with 200 μL of saline intratracheally.

LPS group: Mice received an intratracheal administration of 3 mg/kg LPS once.

Emodin-20 group: Mice were intragastrically administrated with 20 mg/kg emodin once a day for 7 consecutive days. On the last day, mice were treated with 3 mg/kg LPS intratracheally.

Emodin-40 group: Mice were intragastrically administrated with 40 mg/kg emodin once a day for 7 consecutive days. On the last day, mice were treated with 3 mg/kg LPS intratracheally.

SP600125 group: Mice were intraperitoneally injected with 30 mg/kg SP600125 once a day for 7 consecutive days. On the last day, mice were treated with 3 mg/kg LPS intratracheally.

Anisomycin group: Mice were intraperitoneally injected with 15 mg/kg anisomycin. 1 h later, they were intragastrically administrated with 40 mg/kg emodin once a day for 7 consecutive days. On the last day, mice were treated with 3 mg/kg LPS intratracheally.

8 h after the LPS challenge, the mice were sacrificed with administration of 1.5% (*v/v*) sodium pentobarbital. bronchoalveolar lavage fluid (BALF) samples, and lung tissues were collected. BALF samples were used for the determination of TNF-α and IL-6. The superior lobes of the left lung were collected for the wet/dry ratio (W/D) analysis, while the right lobe of the lung was fixed in 4% paraformaldehyde for a histological analysis. The remaining lung tissues were homogenized and stored at –80°C for protein analysis.

### Lung Wet/Dry Weight Ratio Analysis

The fresh lung tissues were excised, blotted dry, and immediately weighted to obtain the wet weight (W). These lung tissues were dried at 60°C in a thermostatic drier (FUMA, Shanghai, China) until the weight did not change and weighted again to obtain the dry weight (D) (Liu et al., 2016). For the evaluation of the degree of pulmonary edema, the W/D ratio was calculated by the following formula: W/D × 100%.

### BALF Preparation and Examination

After the sacrifice of mice, the trachea was separated and then inserted in a plastic cannula. Subsequently, the BALF was collected with two equals of 0.5 ml of cold PBS instilled up to a total volume of 1.0 ml and centrifuged at 500 ×*g* for 10 min at 4°C (Liu et al., 2016). The concentration of total protein in the supernatant of the BALF was measured by a BCA Assay Kit.

### Histological Analysis

The right lobes of lung tissues were isolated, rinsed with cold saline, and fixed in 4% (*w/v*) paraformaldehyde overnight at RT. After being dehydrated and embedded in paraffin, the lung tissues were cut into 5 μm thin sections and stained with hematoxylin and eosin (H&E, Yuanye Biotech, Shanghai, China) (Liu et al., 2016). Tissue sections were observed by using an IX 53 light microscope (Olympus, Tokyo, Japan).

### Statistical Analysis

All data were expressed as mean ± SEM. ANOVA was used to assess differences between multiple groups by SPSS 20.0 (IBM, Armonk, USA). Tukey’s test was used for multiple comparisons. *p* < 0.05 was considered statistically significant.

## Results

### Emodin Inhibits Inflammatory Response and Oxidative Stress *In Vitro* and *In Vivo*

**FIGURE 1:**
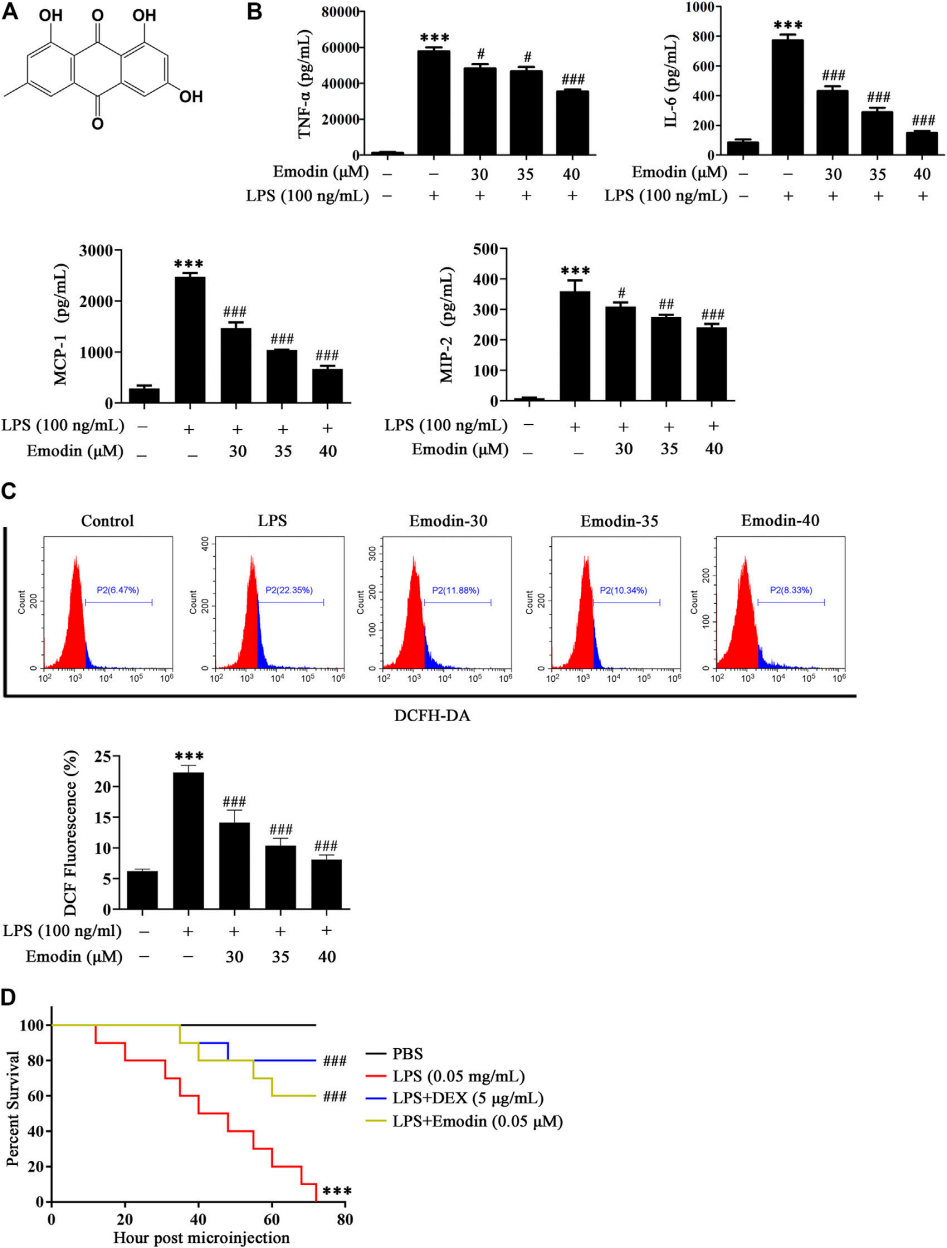
Emodin suppresses inflammatory response and oxidative stress *in vitro* and *in vivo*. **(A)** The chemical structure of emodin. **(B)** Emodin inhibits the production of inflammatory factors TNF-α, IL-6, MCP-1, and MIP-2 in LPS-induced RAW264.7 cells. RAW264.7 cells were pretreated with emodin (30, 35, or 40 μM) for 2 h and then treated with LPS (100 ng/ml) for 18 h. The levels of TNF-α, IL-6, MCP-1, and MIP-2 in supernatant were measured by ELISA. **(C)** Emodin inhibits oxidative stress in LPS-induced RAW264.7 cells. RAW264.7 cells were pretreated with emodin (30, 35, or 40 μM) for 2 h and then treated with LPS (100 ng/ml) for 18 h. The cells were collected and the ROS production was determined by flow cytometric analysis. Data are presented as mean ± SEM, *n* = 3. ^***^ *p* < 0.001 compared with the control group, ^###^ *p* < 0.001 compared with the LPS group. **(D)** Protective effects of emodin on zebrafish challenged by LPS. 3 dpf zebrafish larvae were yolk-microinjected with LPS (0.5 mg/ml), and then treated with emodin (0.05 μM) or DEX (5 μg/ml). Survival of zebrafish larvae was assessed within 72 h. PBS-microinjected zebrafish larvae were served as the negative control. Data are presented as mean ± SEM. ^***^ *p* < 0.001 compared with the PBS group, ^###^ *p* < 0.001 compared with the LPS group.

We first determined the cytotoxicity of emodin in murine macrophage RAW264.7 cells and found that no toxic effects were observed up to 40 μM (Supplementary Figure S1). The chemical structure of emodin is shown in Figure 1A. We therefore used emodin with the dosages of 30–40 μM for further study. Then we determined whether emodin could affect the inflammatory response and oxidative stress in LPS-stimulated RAW264.7 cells. As shown in Figure 1B, after LPS stimulation, the levels of inflammatory factors TNF-α, IL-6, MCP-1, and MIP-2 in the culture medium significantly increased, while emodin pretreatment dose-dependently decreased the secretion of TNF-α, IL-6, MCP-1, and MIP-2.

In addition, a flow cytometric analysis was used to assay the level of ROS. As shown in Figure 1C, the fluorescence intensity of DCF represents the level of intracellular ROS. LPS stimulation significantly accelerated the ROS production compared with the control group. Nevertheless, emodin repressed the secretion of ROS in a dose-dependent manner.

We then determined whether emodin could protect against LPS-induced death in zebrafish larvae. As shown in Figure 1D, all zebrafish larvae challenged with LPS died within 72 h. However, the survival rate of zebrafish larvae that were treated with emodin remarkably increased, and a similar result was observed in DEX-treated larvae.

### Emodin Suppresses the Activation of JNK Signaling in LPS-Stimulated RAW264.7 Macrophage Cells

**FIGURE 2:**
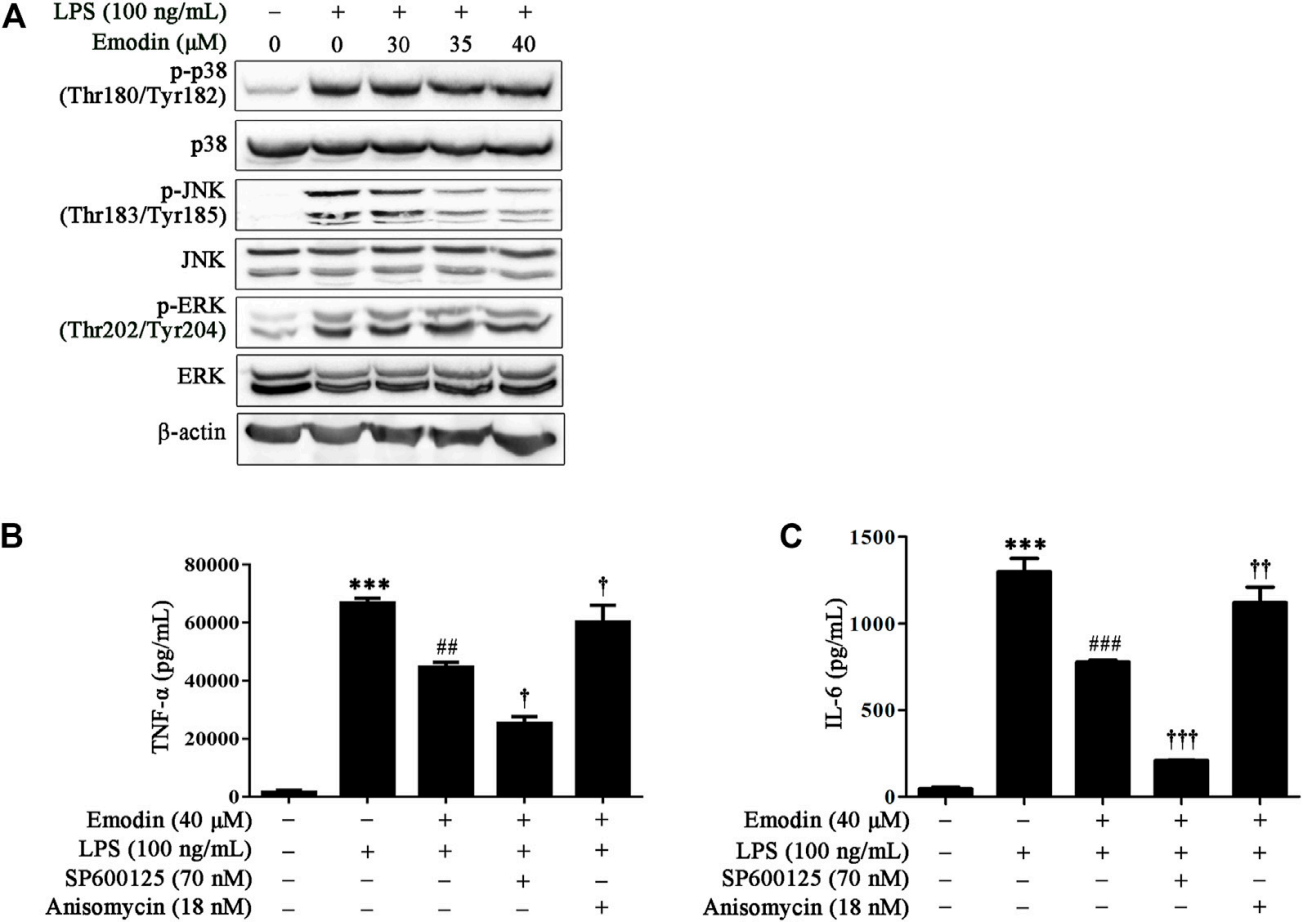
Emodin inhibits LPS-induced inflammation *via* the JNK pathway in RAW264.7 cells. **(A)** Emodin suppresses activation of the JNK pathway in LPS-stimulated RAW264.7 cells. RAW264.7 cells were pretreated with emodin (40 μM) for 2 h followed by exposure to LPS (100 ng/ml) for 18 h. Then the expression levels of p-p38 (Thr180/Tyr182), p38, p-JNK (Thr183/Tyr185), JNK, p-ERK (Thr202/Tyr204), and ERK were assessed using Western blotting. **(B,C)** Anisomycin counteracts the inhibitory effects of emodin on the secretion of inflammatory cytokines. RAW264.7 cells were pretreated with SP600125 (8 μM) for 2 h or anisomycin (18 nM) for 4 h, and then treated with emodin (40 μM) for 2 h followed by exposure to LPS (100 ng/ml) for another 18 h. The levels of TNF-α **(B)** and IL-6 **(C)** in supernatant were measured by ELISA. Data are presented as mean ± SEM, *n* = 3. ^***^ *p* < 0.001 compared with the control group; ^##^ *p* < 0.01, ^###^ *p* < 0.001 compared with the emodin group; ^†^ *p* < 0.05, ^††^ *p* < 0.01, ^†††^ *p* < 0.001 compared with the emodin group.

MAPK signaling plays a crucial role in regulating inflammatory molecules and immune-related pro-inflammatory cytokines (Kaminska et al., 2009; Kyriakis and Avruch, 2012). Therefore, we investigated whether MAPK was involved in the anti-inflammatory effects of emodin in RAW264.7 cells. As shown in Figure 2A, LPS stimulation activated MAPK signaling by increasing the expression levels of p-p38 (Thr180/Tyr182), p-JNK (Thr183/Tyr185), and p-ERK (Thr202/Tyr204). Emodin treatment dose-dependently suppressed LPS-induced JNK phosphorylation, whereas it had no obvious effects on p38 ^Thr180/Tyr182^ and ERK ^Thr202/Tyr204^.

We further investigated whether emodin reduced the secretion of TNF-α and IL-6 due to JNK inhibition; a specific JNK inhibitor, SP600125, was used to block or abolish the expression of p-JNK, whereas anisomycin, a selective JNK activator, was used to upregulate the expression level of p-JNK. Our results showed that SP600125 and anisomycin did not influence the expression of TNF-α and IL-6 in unstimulated RAW264.7 cells (Supplementary Figure S2). However, SP600125 enhanced the inhibitory effect of emodin on TNF-α and IL-6, whereas this inhibitory effect could be counteracted by anisomycin in LPS-stimulated RAW264.7 cells (Figures 2B,C). Taken together, these results indicate that emodin inhibited TNF-α and IL-6 expression by inhibiting JNK signaling in LPS-stimulated RAW264.7 cells.

### Emodin Regulates the Nur77/c-Jun Pathway in LPS-Stimulated RAW264.7 Cells by Inhibiting the JNK Pathway

**FIGURE 3:**
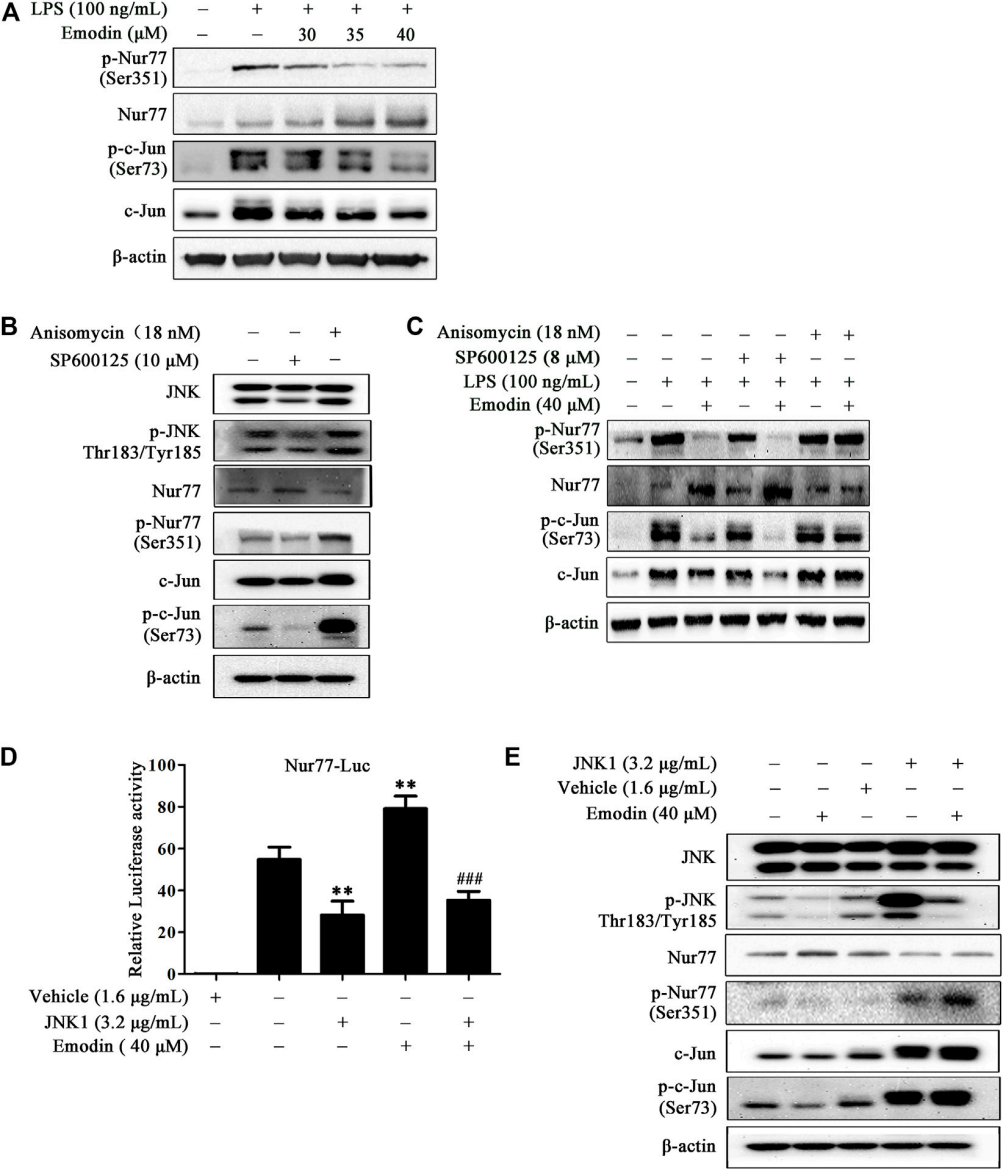
Emodin regulates the Nur77/c-Jun pathway in LPS-stimulated RAW264.7 cells by inhibiting the JNK pathway. **(A)** Emodin regulates the Nur77/c-Jun pathway. RAW264.7 cells were pretreated with emodin (40 μM) for 2 h followed by exposure to LPS (100 ng/ml) for 18 h. The expression levels of p-Nur77 (Ser351), Nur77, p-c-Jun (Ser73), and c-Jun were measured by Western blotting. **(B)** The functional verification of SP600125 and anisomycin. RAW264.7 cells were treated with SP600125 (10 μM) for 22 h or anisomycin (18 nM) for 24 h. The expression levels of p-JNK (Thr183/Tyr185), JNK, p-Nur77 (Ser351), Nur77, p-c-Jun (Ser73), and c-Jun in total cell lysates were detected by Western blotting. **(C)** Anisomycin blocks the regulatory effects of emodin on the Nur77/c-Jun pathway. RAW264.7 cells were pretreated with SP600125 (8 μM) for 2 h or anisomycin (18 nM) for 4 h, and then treated with emodin (40 μM) for 2 h, followed by exposure to LPS (100 ng/ml) for another 18 h. The expression levels of p-Nur77 (Ser351), Nur77, p-c-Jun (Ser73), and c-Jun in total cell lysates were detected by Western blotting. **(D)** Emodin increases the transcriptional activity of Nur77 *via* the JNK pathway. HEK293.7T cells were transfected with plasmid Nur77-luciferase (1.6 μg/ml) and pRL-TK (1.6 μg/ml) in the presence or absence of JNK1 plasmid (3.2 μg/ml). After 24 h transfection, cells were treated with emodin for 8 h, and then luciferase assay was performed. Relative luciferase activity was expressed as the ratio of firefly to Renilla luciferase. Data are presented as mean ± SEM, *n* = 3. **p < 0.01 compared with the control group; ^###^ *p* < 0.001 compared with the emodin group. **(E)** Emodin increases the protein expression of Nur77 *via* the JNK pathway. HEK293.7T cells were transfected with JNK1 plasmid (3.2 μg/ml). After 24 h transfection, cells were treated with emodin for 8 h, and then the expression levels of *p*-JNK (Thr183/Tyr185), JNK, p-Nur77 (Ser351), Nur77, p-c-Jun (Ser73), and c-Jun in total cell lysates were assessed using Western blotting.

Since the orphan nuclear receptor Nur77 was recognized as an important regulator in inflammation, the suppression of c-Jun was reported to inhibit inflammatory response *in vitro* and in the LPS-induced ALI mice model (Lee et al., 2016; Liu et al., 2017; Yang et al., 2018). Moreover, it has been shown that the interaction of Nur77 and c-Jun inhibits the c-Jun promoter activity, decreasing the expression of c-Jun involved in AP-1 transcriptional activity and endothelin-1 expression in human umbilical vein endothelial cells (Qin et al., 2014). We further explored the effect of emodin on Nur77 and c-Jun in LPS-stimulated RAW264.7 cells. As shown in Figure 3A, on LPS stimulation, Nur77 was phosphorylated at Ser351 and c-Jun was phosphorylated at Ser73, and the expression levels of Nur77 and c-Jun were also elevated. However, emodin dose-dependently inhibited the phosphorylation of Nur77 and c-Jun, upregulated the expression level of Nur77, and downregulated the expression level of c-Jun.

To further elucidate the role of JNK in the anti-inflammatory effects of emodin, JNK inhibitor SP600125 and activator anisomycin were employed. First, we performed functional verification on SP600125 and anisomycin (Figure 3B). Our results showed that SP600125 (10 μM) could inhibit the expression of p-JNK (Thr183/Tyr185), and anisomycin (18 nM) increased p-JNK expression in RAW264.7 cells. Moreover, compared to the control group, SP600125 inhibited the expression of p-Nur77 (Ser351), p-c-Jun (Ser73), and c-Jun, and increased the expression of Nur77. Nevertheless, anisomycin increased the protein expression of p-Nur77 (Ser351), p-c-Jun (Ser73), and c-Jun, reducing the Nur77 expression. To better explore the coordination effects of emodin and SP600125, we chose a lower dose of SP600125 (8 μM) for follow-up research. Similarly, as shown in Figure 3C, blockage of JNK by SP600125 enhanced the inhibitory effects of emodin on phosphor-c-Jun, the expression levels of c-Jun, and the upregulatory effect of emodin on Nur77 expression in LPS-stimulated RAW264.7 cells. However, the activation of JNK by anisomycin counteracted the regulatory effects of emodin on the Nur77/c-Jun signaling pathway.

Moreover, in HEK293T cells, JNK overexpressed plasmid was used to up-regulate the JNK expression (Supplementary Figure S3), and the overexpression of JNK inhibited the Nur77 luciferase activity induced by emodin (Figure 3D). Similarly, Western blotting results showed that JNK overexpressed plasmid counteracted the inhibitory effects of emodin on p-JNK (Thr183/Tyr185), p-c-Jun (Ser73), and p-Nur77 (Ser351), and the upregulatory effects of Nur77 (Figure 3E). Taken together, these results demonstrate that the regulation of emodin on Nur77 and c-Jun signaling is associated with JNK inhibition.

### Emodin Suppresses LPS-Induced Activation of c-Jun Through Regulation of Nur77

**FIGURE 4:**
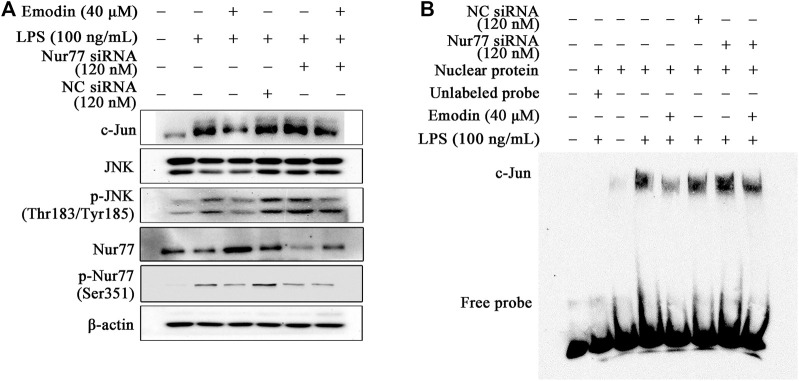
Emodin suppresses LPS-induced activation of c-Jun through regulation of Nur77. **(A)** Emodin decreases the expression levels of p-c-Jun (Ser73) and c-Jun through regulation of Nur77. RAW264.7 cells were transfected with Nur77 siRNA (120 nM) for 24 h, and then treated with emodin (40 μM) for 2 h, followed by exposure to LPS (100 ng/ml) for another 18 h, p-c-Jun (Ser73), and c-Jun were detected by Western blotting. **(B)** Emodin inhibits the DNA-binding activity of c-Jun through regulation of Nur77. The nuclear lysates were harvested and the DNA-binding activity were measured using EMSA.

It has been proven that Nur77 decreased c-Jun expression because of inhibiting the AP-1 dependent c-Jun promoter activity (Qin et al., 2014). To study whether Nur77 regulates c-Jun activity in emodin-mediated anti-inflammatory activity, Nur77 siRNA was applied to silence the expression of Nur77 (Supplementary Figure S4), and then the expression of c-Jun was assayed. It could be found that the inhibitory effect of emodin on the total c-Jun expression was counteracted by Nur77 siRNA (Figure 4A). In addition, EMSA results suggested that Nur77 siRNA counteracted the inhibitory effects of emodin on the DNA-binding activity of c-Jun (Figure 4B). Taken together, these results indicate that emodin suppresses the activation of c-Jun *via* Nur77 signaling.

### Emodin Protects Against LPS-Induced ALI Mice Through Inhibition of the JNK Signaling

**FIGURE 5:**
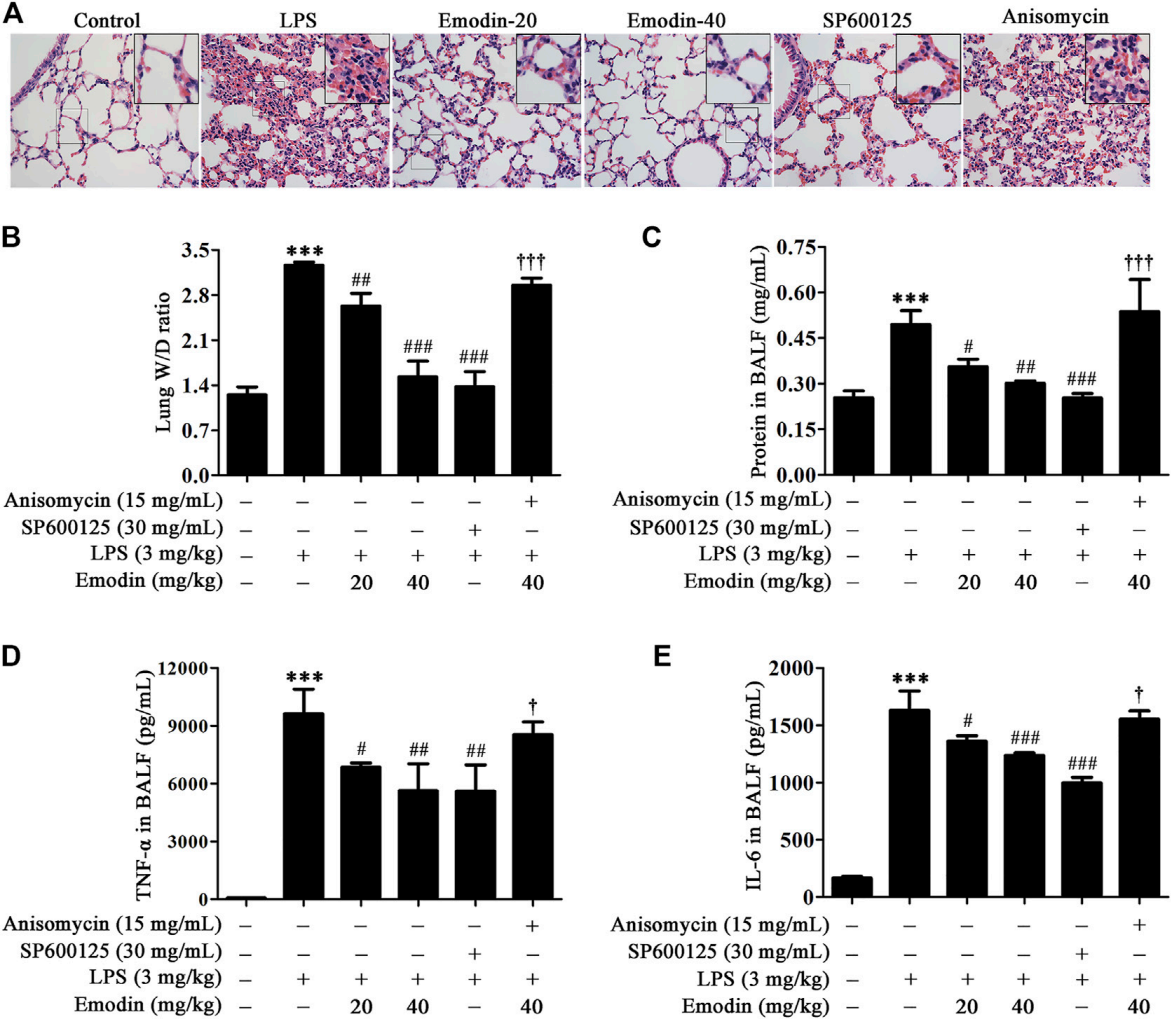
Emodin inhibits LPS-induced acute lung injury (ALI) in mice *via* the JNK pathway. **(A)** SP600125 alleviates the protective effects of emodin on lung histopathological changes in ALI mice. The lung tissues were stained with hematoxylin and eosin (H&E) (40 ×). (**B–E)** Anisomycin counteracts the inhibitory effects of emodin on inflammation and edema in ALI mice. At 8 h after stimulation by LPS, lung tissues were separated and then weighted to obtain the W/D ratio **(B)**. BALF was collected to detect the total protein concentration **(C)** using the BCA protein assay kit, and the levels of TNF-α **(D)** and IL-6 **(E)** in the supernatant were measured by ELISA. Data are presented as mean ± SEM, *n* = 8. ^***^ *p* < 0.001 compared with the control group; ^#^ *p* < 0.05, ^##^ *p* < 0.01, ^###^ *p* < 0.001 compared with the LPS group; ^†^ *p* < 0.05, ^†††^ *p* < 0.001 compared with the emodin-40 group.

Inflammatory response contributes to the progression of ALI. Previous studies have shown that the inhibition of JNK signaling could suppress inflammatory response in animal models, including LPS-induced ALI mice (Ha et al., 2018; Fei et al., 2019). Therefore, we verified whether emodin could alleviate ALI through the JNK signaling pathway. As shown in Figure 5A, edema, hyperemia, and inflammatory cell infiltration were reduced in the lung tissues in emodin-treated ALI mice, compared with LPS-induced ALI mice. In addition, emodin treatment decreased edematous and inflammatory indexes, including the lung W/D ratio (Figure 5B), total protein (Figure 5C), and the expression levels of pro-inflammatory cytokines TNF-α (Figure 5D) and IL-6 (Figure 5E) in BALF. Taken together, emodin at the dosage of 20 and 40 mg/kg possesses remarkable anti-ALI effects. Similar effects were also observed in SP600125-treated mice. However, the protective effects of emodin were counteracted in the presence of anisomycin (15 mg/kg). These data indicate that emodin attenuates inflammation in ALI mice through the inhibition of JNK signaling.

**FIGURE 6:**
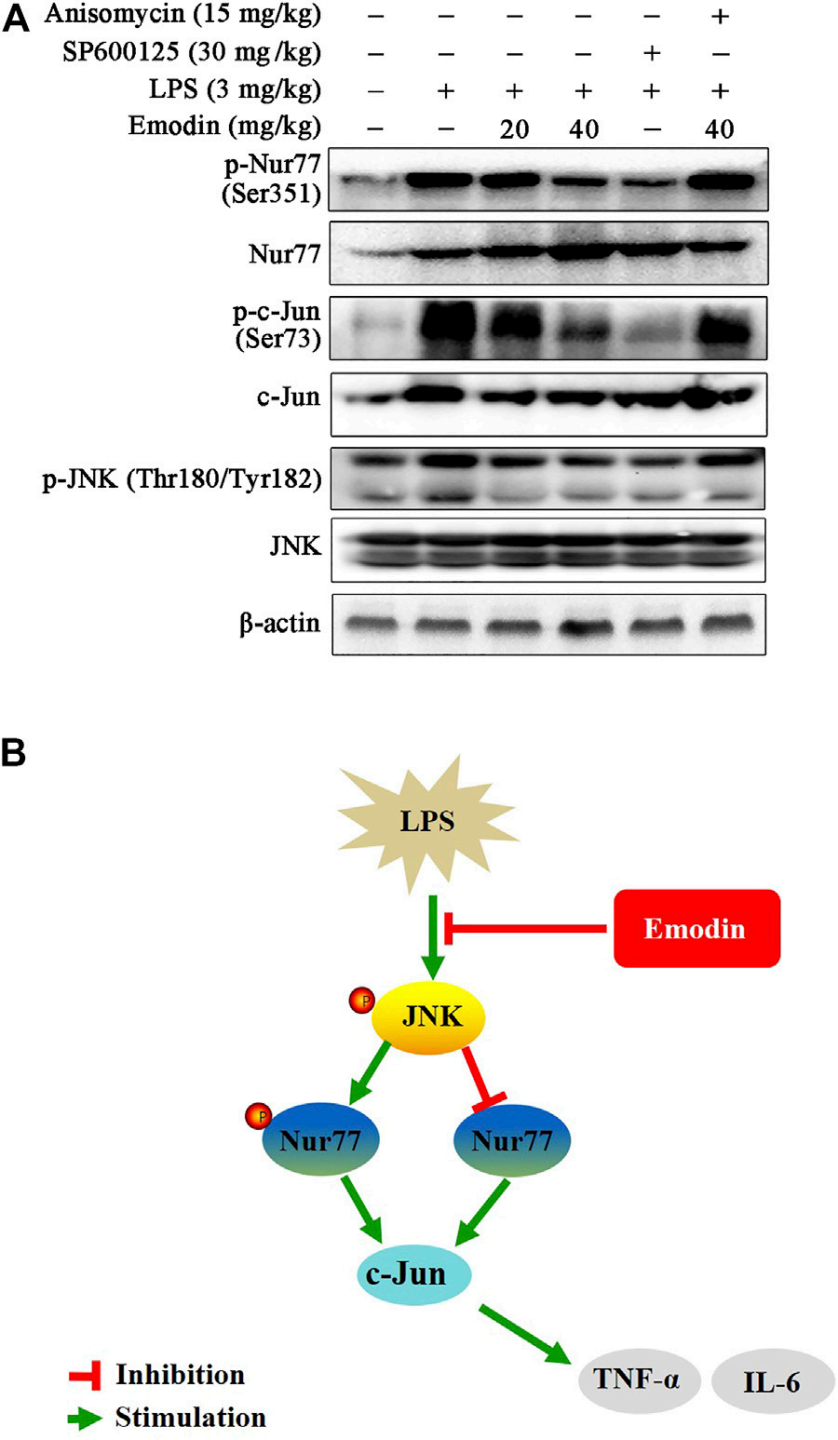
**(A)** Emodin regulates the Nur77/c-Jun pathway through inhibition of the JNK pathway in LPS-induced acute lung injury mice. The lung tissues were harvested and homogenized. The expression levels of P-Nur77 (Ser351), Nur77, p-c-Jun (Ser73), and c-Jun in total lysates were examined using Western blotting. **(B)** Illustration of protection provided by emodin on LPS-induced inflammation.

We further explored whether emodin regulates the activity of Nur77 and c-Jun *via* the JNK signaling pathway in LPS-induced ALI mice. As shown in Figure 6A, emodin treatment dose-dependently increased the expression levels of Nur77, and decreased the levels of phosphor-Nur77, phosphor-c-Jun, and c-Jun in LPS-induced ALI mice. Similar results were also observed in the SP600125 group. Notably, anisomycin could reduce the effects of emodin on the Nur77/c-Jun signaling pathway, indicating that emodin regulates the activity of the Nur77/c-Jun pathway through JNK inhibition in LPS-induced ALI mice.

## Discussion

Dysregulated inflammatory response, diffuse alveolar damage, proteinaceous edema, hyaline membranes, and even fibroplasia are the main pathophysiological characteristics of ALI (Matthay et al., 2003; Elicker et al., 2016). Recent clinical strategies for ALI/ARDS are limited to mechanical ventilation and supportive treatments (Matthay et al., 2017). Therefore, a successful treatment for ALI/ARDS is an urgent clinical challenge. Emodin, an active component extracted from *Rheum palmatum* L., has been shown to exert biological activities in inflammatory and infectious diseases (Kim et al., 2008; Jia et al., 2014). In the last few years, studies have shown that emodin could ameliorate the lung histopathological changes in ALI models (Xiao et al., 2014; Xu et al., 2016; Cui et al., 2017). In this study, our results showed that emodin exerted significant anti-inflammatory properties *in vitro* and *in vivo via* the JNK/Nur77/c-Jun signaling pathway. In this study, the difference in the dosage of emodin between a murine macrophage cell line RAW264.7 and zebrafish models may be due to the different species. To our knowledge, this is the first report of emodin exerting the anti-ALI effects *via* the JNK/Nur77/c-Jun signaling pathway.

A number of studies have proven that the MAPK signaling pathway, including p38, JNK, and ERK, is activated in the LPS-induced mice model of ALI (Yu et al., 2016; Hsieh et al., 2017). Consistent with previous findings, our study showed that LPS activated the MAPK signaling pathway by increasing the phosphorylation of p38 at Thr180/Tyr182, JNK at Thr183/Tyr185, and ERK at Thr202/Tyr204 in RAW264.7 cells. Interestingly, we found that emodin inhibited JNK phosphorylation but had no effect on the expression of JNK, p38 phosphorylation, or ERK phosphorylation. However, previous studies reported that emodin decreased the concanavalin A-induced phosphorylation of p38 in RAW264.7 cells as well as LPS-induced phosphorylation of ERK and p38 in mouse peritoneal macrophages (Jia et al., 2014; Xue et al., 2015). The controversial results of emodin on the MAPK pathway might be due to the different types of inflammation models. Moreover, we found that the inhibitory effects of emodin on the production of TNF-α and IL-6 were exaggerated by JNK blockage, but can be counteracted by JNK activation, which further indicated that the inhibition of the JNK pathway contributed to emodin-mediated anti-inflammatory activity.

Orphan nuclear receptor Nur77 plays an important role in inflammation. Nur77 is upregulated in macrophages in response to pro-inflammatory stimuli including LPS, TNF-α, prostaglandins, and interferon gamma (Bonta et al., 2006; Shao et al., 2010). Nur77 blocks macrophages toward an inflammatory phenotype through the inhibition of inflammatory gene expression and the regulation of chemokine (C-X3-C motif) receptor 1 (CX3CR1) expression (Hamers et al., 2016; Koenis et al., 2018). A study has proven that 5-aminosalicylic acid inhibited monocrotaline-induced inflammatory responses in pulmonary arterial hypertension rats by increasing the Nur77 expression (Sun et al., 2017). Similarly, we found that emodin upregulated Nur77 in LPS-stimulated RAW264.7 macrophage cells and ALI mice. Further studies showed that emodin increased the promoter activity of Nur77 in LPS-stimulated RAW264.7 cells and inhibited LPS-induced phosphorylation of Nur77 at the Ser351 site *in vitro* and *in vivo*. However, a previous study reported that 1 hour after LPS stimulation, phosphorylation of Nur77 at the Th site increased in RAW264.7 cells, but not the Ser site (Li et al., 2015). These different phosphorylation sites activated by LPS might be due to the difference of LPS stimulation time in RAW264.7 cells.

TNF-α induces Nur77 expression by activating JNK, which leads to attenuating the death effect of TNF-α in cancer cells (Xu et al., 2016). Moreover, studies have shown that phosphorylated Nur77 promoted cell apoptosis and inhibition of JNK suppressed the phosphorylation of Nur77, resulting in alleviating early brain injury after a subarachnoid hemorrhage (Dai et al., 2014). The aforementioned studies indicated that Nur77 could regulate cell survival *via* JNK signaling and therefore inhibit the inflammatory response. In our study, we demonstrated that emodin exerted anti-inflammatory effects that resulted from enhancing the promoter activity and expression level of Nur77 as well as inhibiting the Nur77 phosphorylation *via* JNK signaling. Activated JNK can phosphorylate the transcription factor c-Jun, which induces c-Jun nuclear translocation and finally regulates the transcription of target genes that are involved in inflammatory response (Ip and Davis, 1998; Rahman et al., 2002). We speculate that the decrease of c-Jun leads to the reduction of the inflammatory factors TNF-α and IL-6 production. It was reported that polyphenol-rich peanut extract reduced extracellular TNF-α protein by inhibiting c-Jun transcription factor activity, suggesting an anti-inflammatory effect (Catalán et al., 2012). Moreover, *in vivo* and *in vitro* inhibition of c-Jun could reduce H5N1 influenza virus replication and subsequent inflammatory reactions (Xie et al., 2014). Similarly, our results showed that emodin downregulated the expression level of c-Jun in LPS-treated RAW264.7 cells and ALI models. In addition, emodin also suppressed c-Jun phosphorylation at Ser73 in this study. Studies have demonstrated that emodin decreased the expression of c-Jun due to the reduction of adenosine 5′-monophosphate (AMP)-activated protein kinase-alpha and the inhibition of COP9 signalosome-associated kinases, which contributed to the anti-cancer effects of emodin (Füllbeck et al., 2005; Tang et al., 2015). But the inhibitory effects of emodin on c-Jun expression in inflammatory responses have not been reported so far. Furthermore, activation of JNK counteracted the inhibitory effects of emodin on c-Jun, indicating that emodin repressed the activation of c-Jun *via* JNK signaling in inflammatory responses. Taken together, previous findings and our aforementioned data suggest that emodin inhibits both Nur77 and c-Jun phosphorylation and increases Nur77 expression, suggesting that Nur77 and c-Jun may be JNK downstream.

A previous study suggested that c-Jun combined with the promoter of Nur77, resulting in an increase in Nur77 expression which promotes the production of testosterone steroids (Lee et al., 2009). Moreover, the combination of Nur77 and c-Jun inhibits the expression of c-Jun, which results in a decrease of endothelin-1 in vascular endothelial cells (Qin et al., 2014). The previously mentioned studies suggested there was mutual regulation between Nur77 and c-Jun. Our data also demonstrated that the blockage of Nur77 abolished the inhibitory effects of emodin on c-Jun expression in RAW264.7 cells. These results may be explained by the increased Nur77 levels in the nucleus, which may increase the chance of Nur77 binding to c-Jun, leading to a decreased c-Jun expression. In addition, in this study, emodin also suppressed c-Jun phosphorylation at Ser73. These findings and our results suggest that emodin has multiple effects on c-Jun. Emodin may inhibit the expression of c-Jun due to the binding of Nur77 to c-Jun; on the other hand, it may inhibit c-Jun phosphorylation due to JNK inhibition.

Recently, coronavirus disease 2019 (COVID-19), a declared global pandemic caused by severe acute respiratory syndrome corona virus 2 (SARS-CoV-2), is characterized by an excessive inflammatory response (Huang et al., 2020). Cytokine storms including pro-inflammatory cytokines and chemokine response lead to the deterioration and death of patients with COVID-19 (Ye et al., 2020). A previous study showed that emodin could inhibit the interaction of the SARS-CoV S protein and angiotensin converting enzyme 2 (ACE2) (Ho et al., 2007). A recent study found that SARS-CoV-2 and SARS-CoV had a 79.6% sequence identity and the same cell entry receptor (ACE2) (Zhou et al., 2020). These studies indicate that emodin may be a potential therapeutic agent to treat COVID-19.

## Conclusion

Our study shows that emodin effectively inhibits LPS-induced inflammatory response in RAW264.7 macrophage cells, zebrafish model, and ALI mice. Emodin suppresses LPS-induced JNK activation, and subsequently regulates the downstream mediators Nur77 and c-Jun, which results in the reduction of inflammatory cytokines. Moreover, emodin inhibits the activity of c-Jun through the upregulation of Nur77 (Figure 6B). Therefore, the JNK/Nur77/c-Jun pathway may be a potential target for developing therapies for ALI, and emodin may have the utility as an anti-ALI agent.

## Data Availability

The raw data supporting the conclusions of this article will be made available by the authors, without undue reservation.
