# Supplementary material for: Emodin Protects Against Lipopolysaccharide-Induced Acute Lung Injury via the JNK/Nur77/c-Jun Signaling Pathway
Source: Front Pharmacol. 2022 Mar 17;13:717271. doi: 10.3389/fphar.2022.717271 (PMC8968870; doi:10.3389/fphar.2022.717271)
Supplement: Supplementary file 1 [file DataSheet4.docx]

Supplementary Material


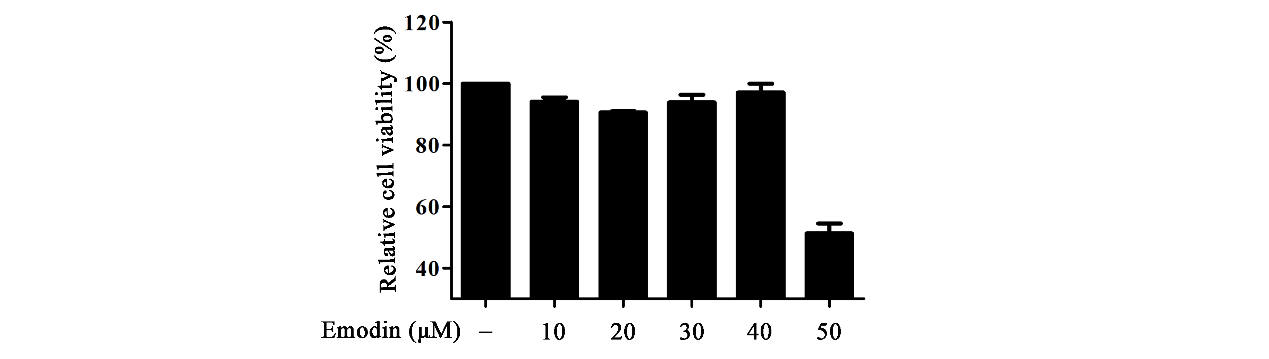


**Supplementary Figure 1.** The cytotoxicity of emodin in RAW264.7 cells. RAW264.7 cells were treated with indicated concentrations of emodin for 24 h. The cell viability was determined using MTT assay. Data are presented as mean ± SEM, n=3.


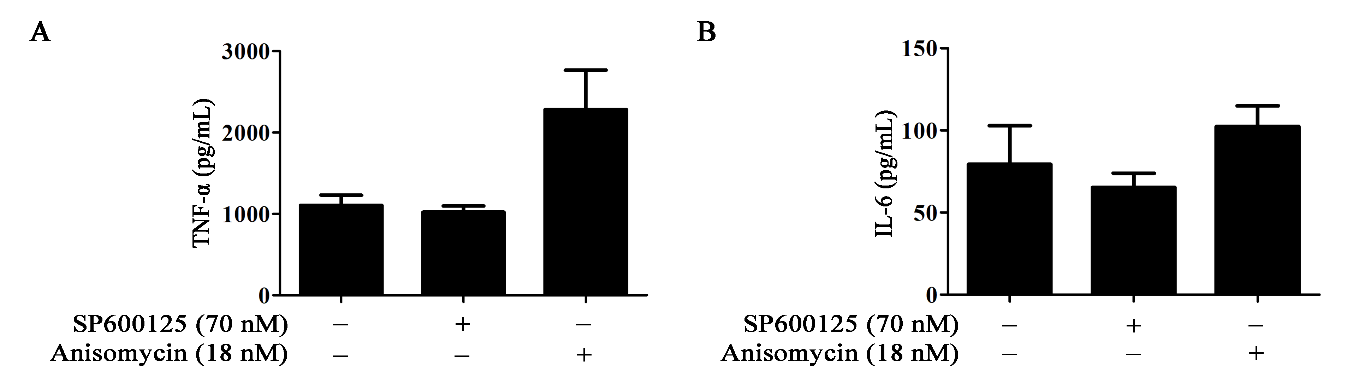


**Supplementary Figure 2.** Effects of SP600125 and anisomycin on the production of pro-inflammatory cytokines in RAW264.7 cells. RAW264.7 cells were treated with SP600125 (8 μM) for 22 h or anisomycin (18 nM) for 24 h. The levels of TNF-α (A) and IL-6 (B) in supernatant were measured by ELISA. Data are presented as mean ± SEM, n=3.


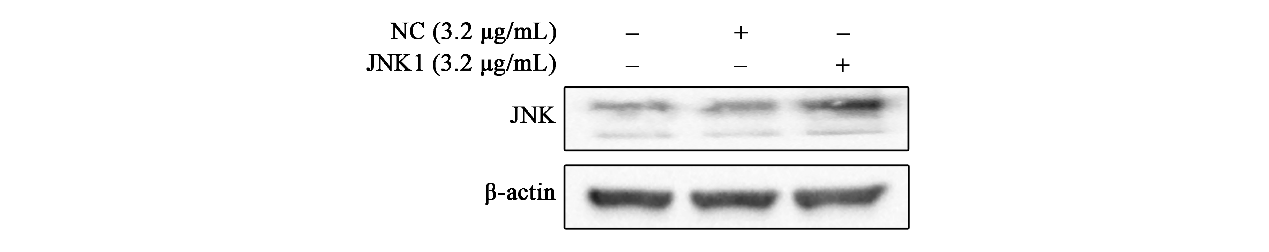


**Supplementary Figure 3.** The functional verification of JNK1 overexpressed plasmid. HEK293.7T cells were transfected with a JNK1 overexpressed plasmid (3.2 μg/mL) for 24 h. The expression level of JNK in total lysates was measured using Western blotting.


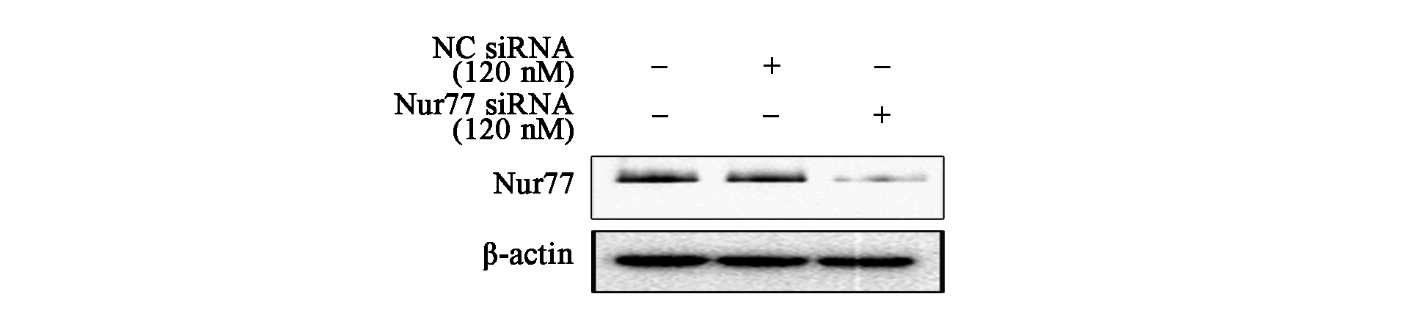


**Supplementary Figure 4.** The functional verification of Nur77 siRNA. RAW264.7 cells were transfected with Nur77 siRNA for 44 h. Total cell lysates were subjected to Western blotting for measuring the expression of Nur77.
