# Supplementary figures and images for: Emodin Protects Against Lipopolysaccharide-Induced Acute Lung Injury via the JNK/Nur77/c-Jun Signaling Pathway
Source: Front Pharmacol. 2022 Mar 17;13:717271. doi: 10.3389/fphar.2022.717271 (PMC8968870; doi:10.3389/fphar.2022.717271)

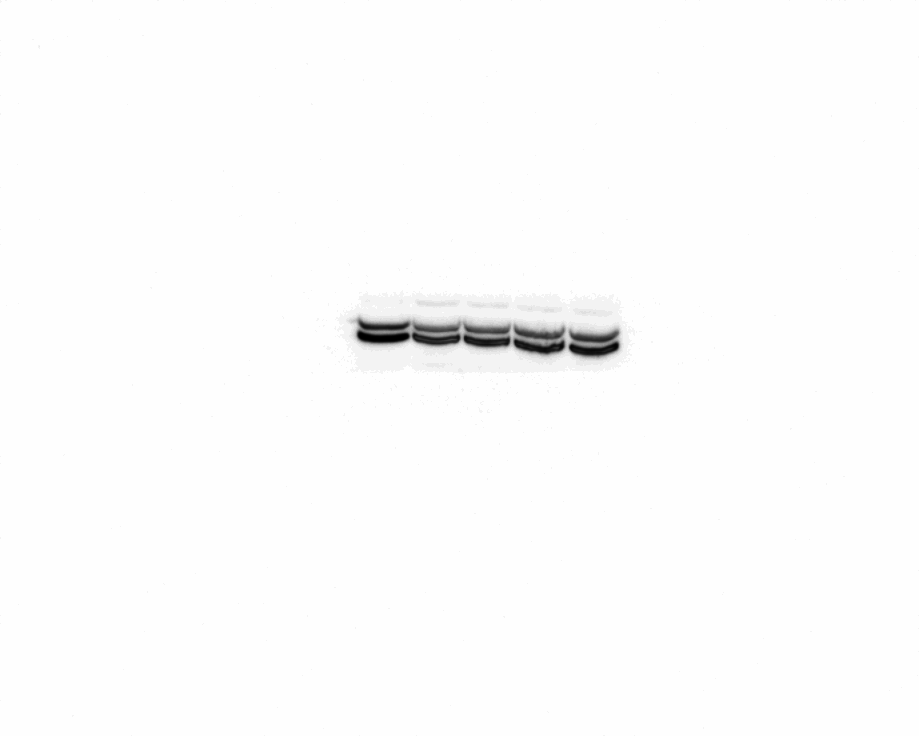

Supplement: Supplementary file 3 [file DataSheet1.ZIP › Original gels/Figure 2/ERK.tif]

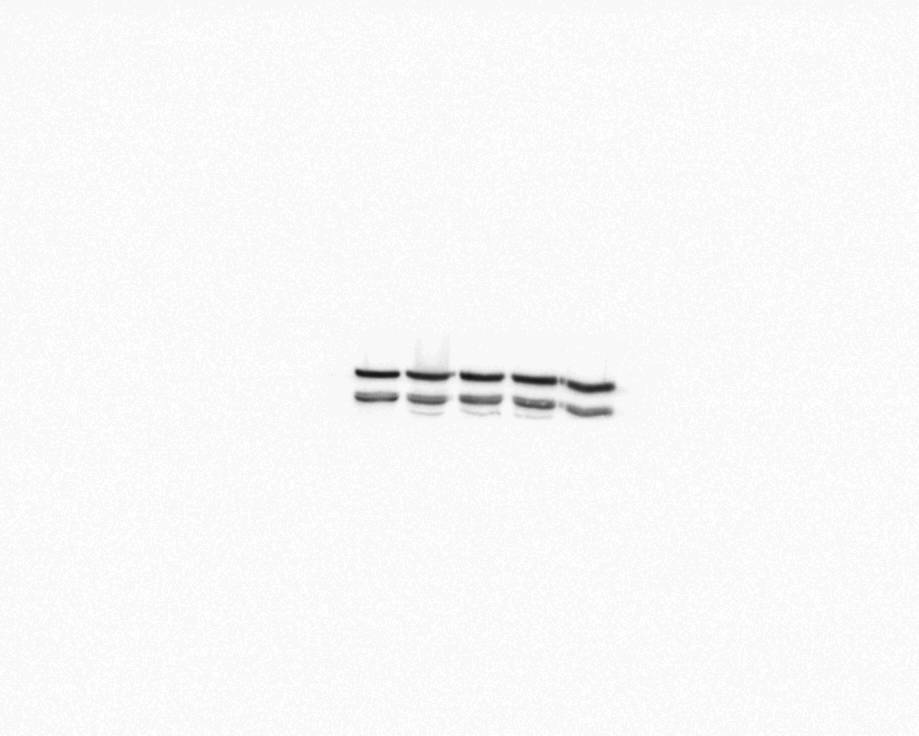

Supplement: Supplementary file 3 [file DataSheet1.ZIP › Original gels/Figure 2/JNK.tif]

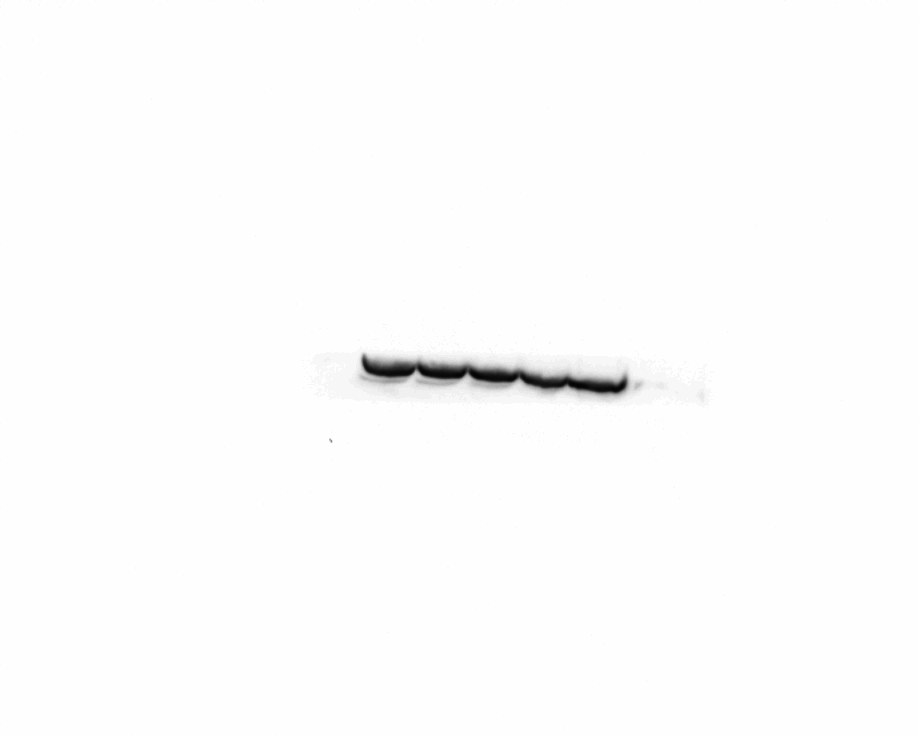

Supplement: Supplementary file 3 [file DataSheet1.ZIP › Original gels/Figure 2/P38.tif]

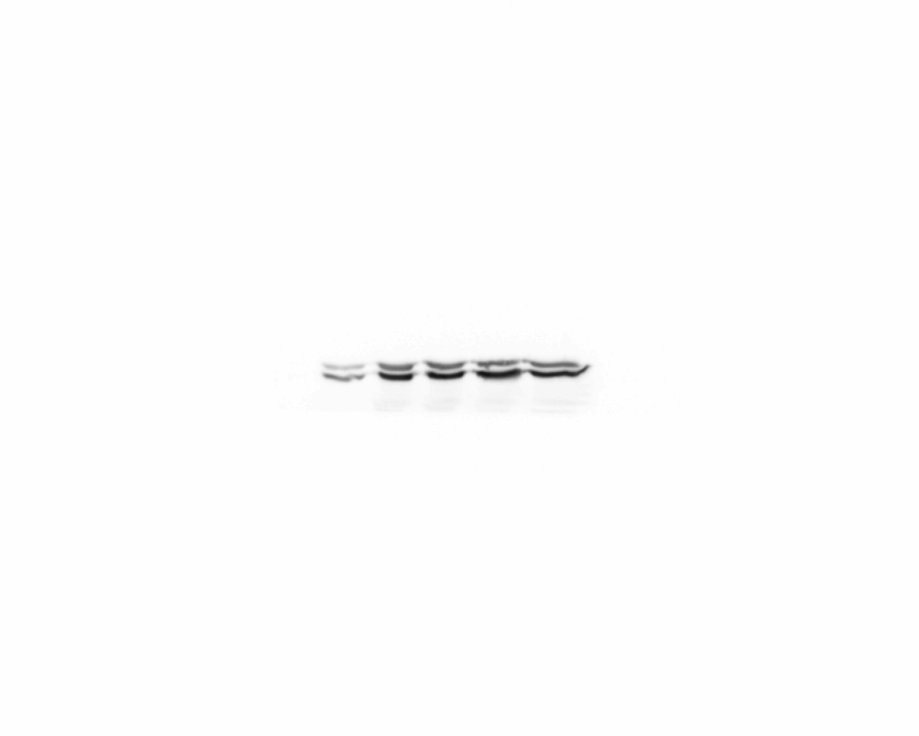

Supplement: Supplementary file 3 [file DataSheet1.ZIP › Original gels/Figure 2/p-ERK.tif]

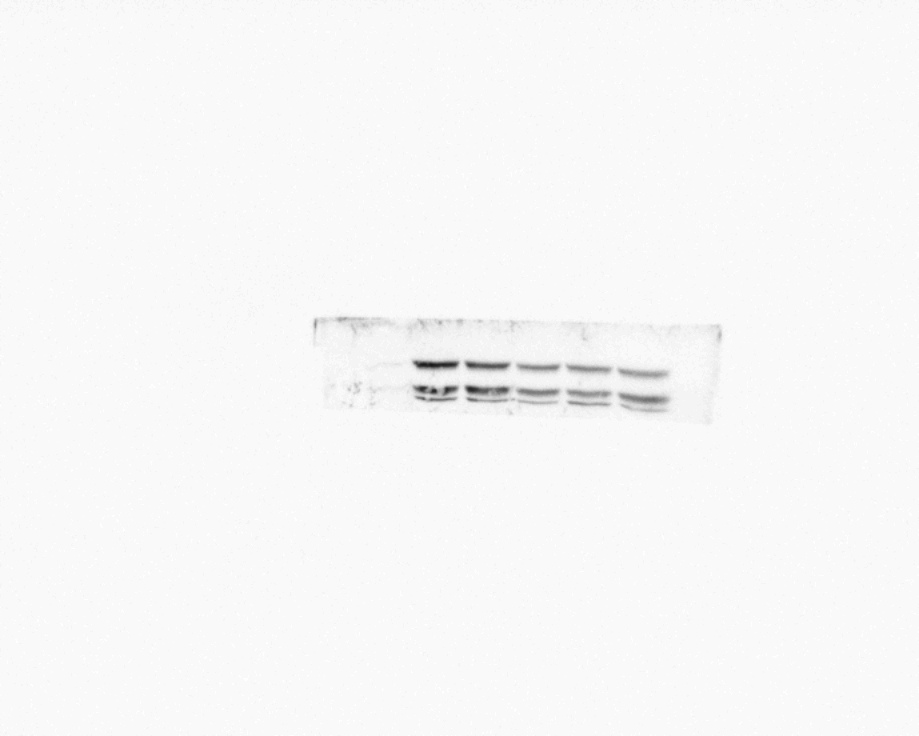

Supplement: Supplementary file 3 [file DataSheet1.ZIP › Original gels/Figure 2/p-JNK.tif]

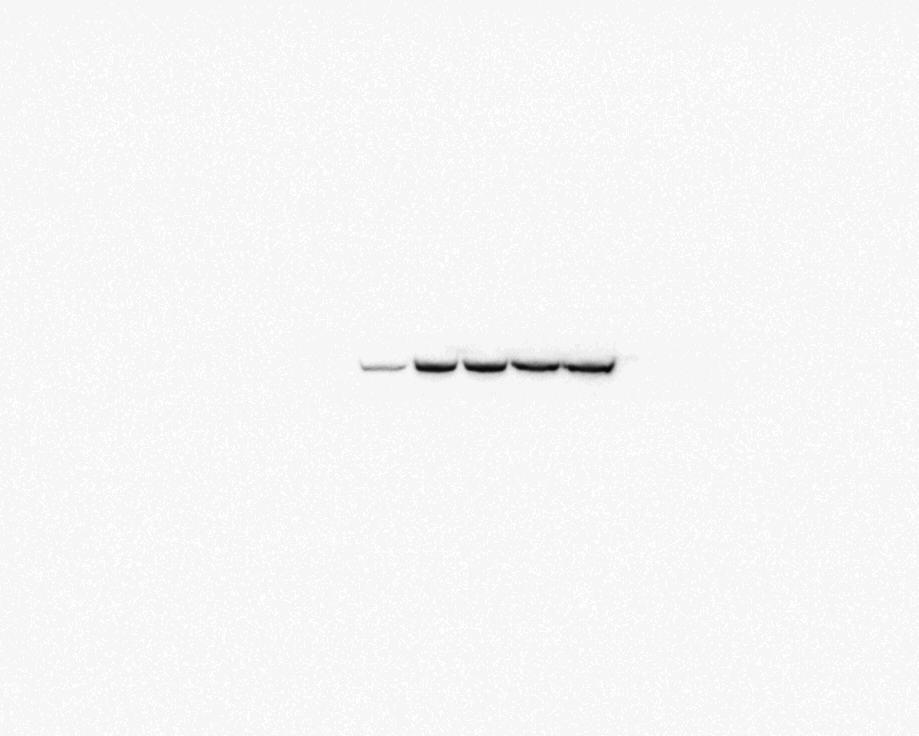

Supplement: Supplementary file 3 [file DataSheet1.ZIP › Original gels/Figure 2/p-p38.tif]

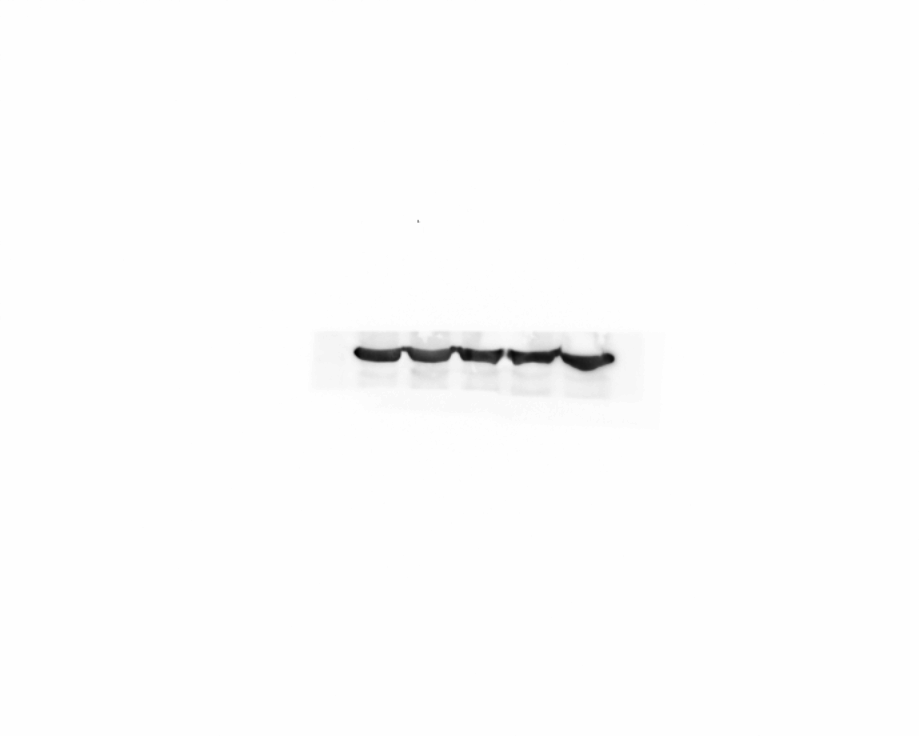

Supplement: Supplementary file 3 [file DataSheet1.ZIP › Original gels/Figure 2/a┬-actin.tif]

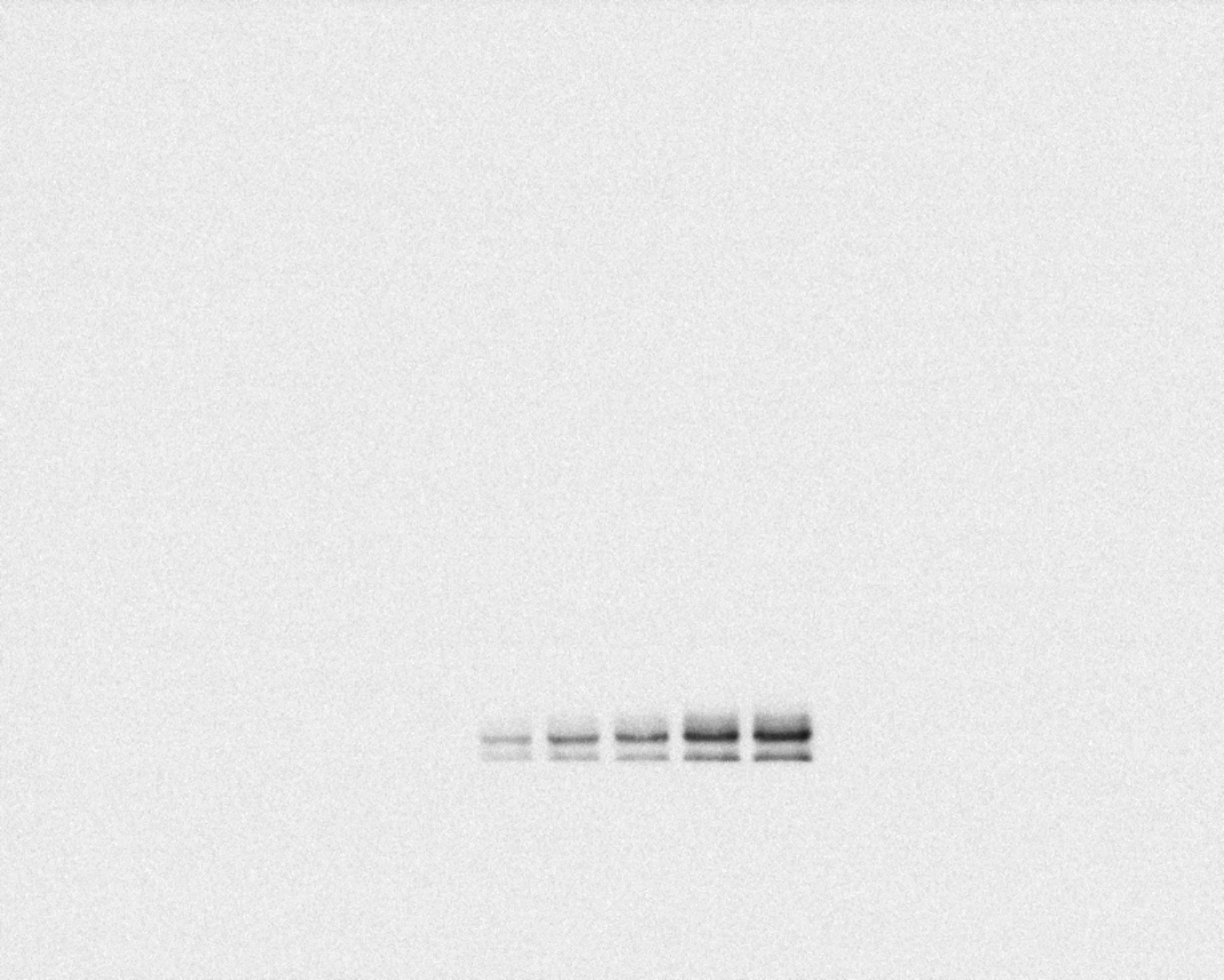

Supplement: Supplementary file 3 [file DataSheet1.ZIP › Original gels/Figure 3A/Nur77.tif]

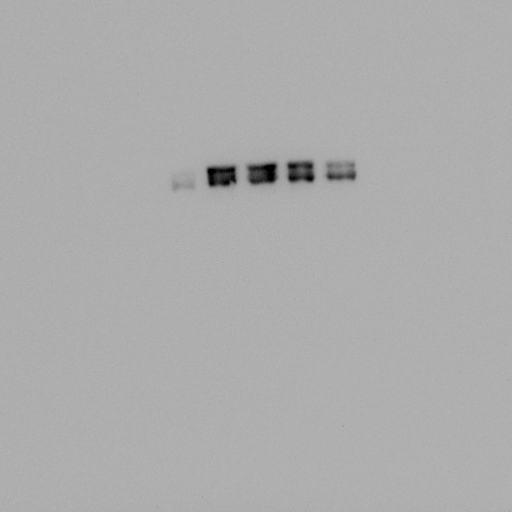

Supplement: Supplementary file 3 [file DataSheet1.ZIP › Original gels/Figure 3A/p-c-Jun.tif]

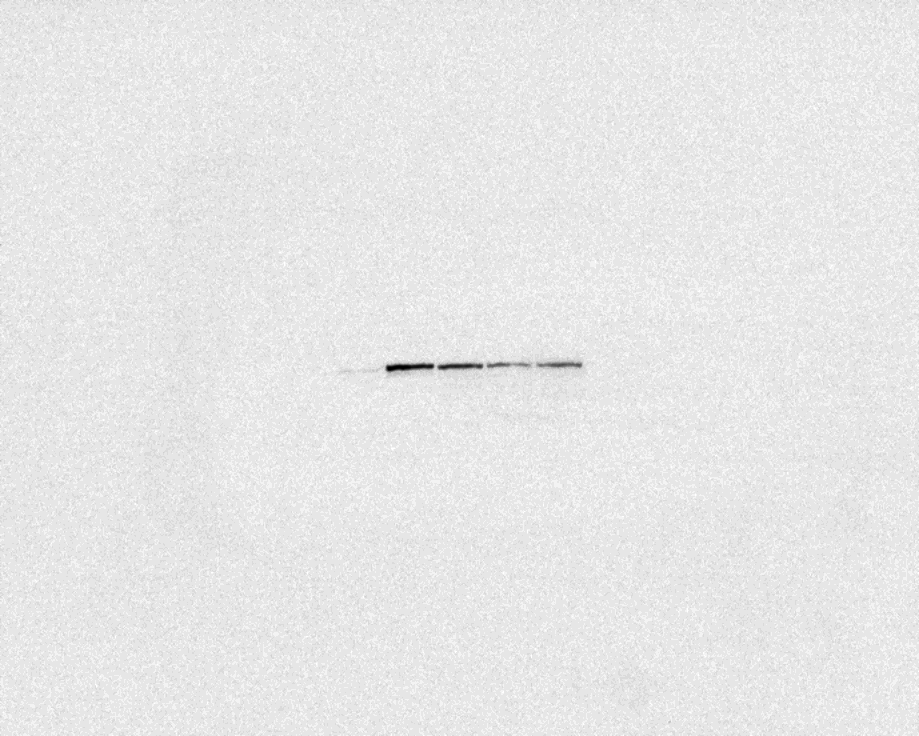

Supplement: Supplementary file 3 [file DataSheet1.ZIP › Original gels/Figure 3A/p-Nur77.tif]

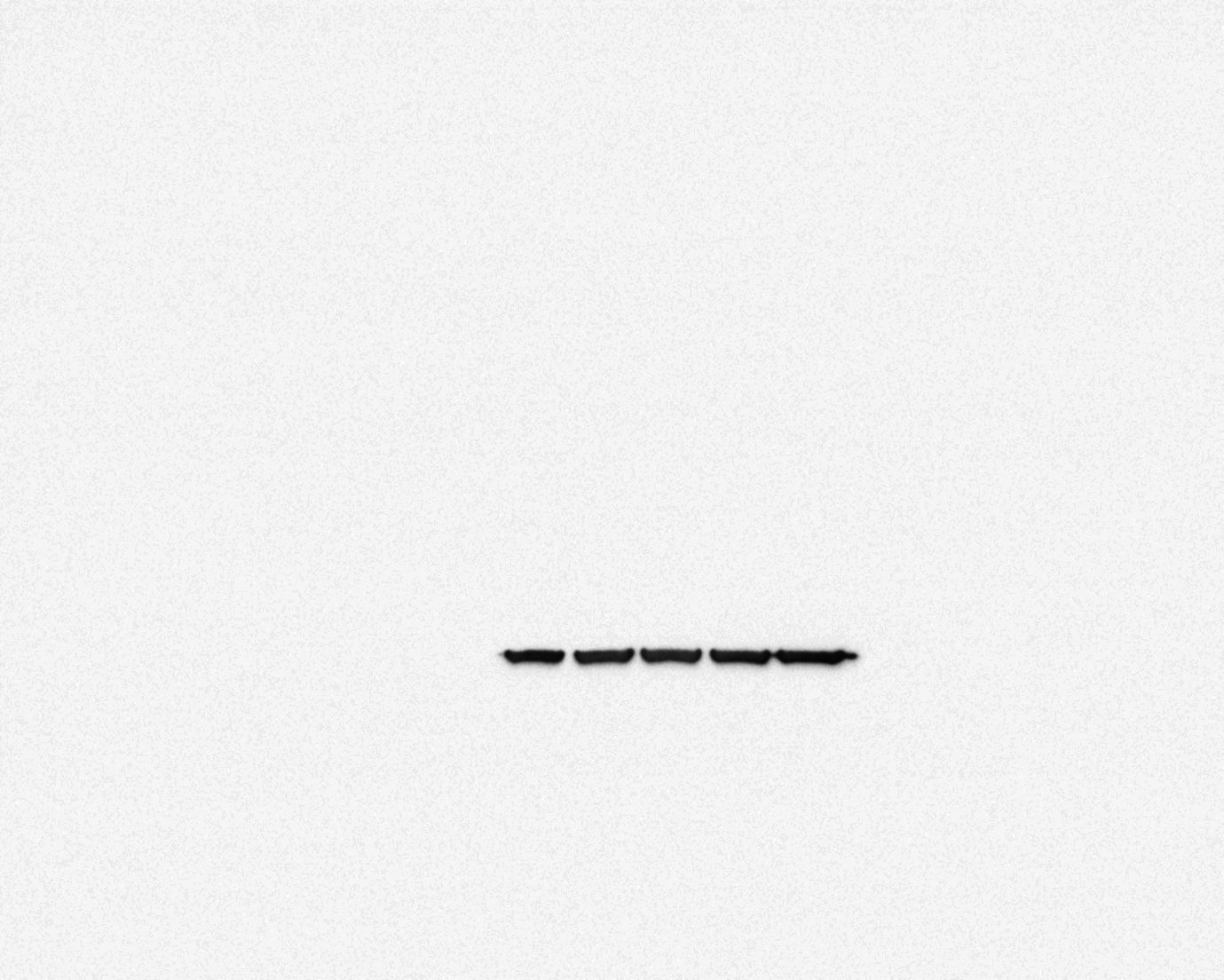

Supplement: Supplementary file 3 [file DataSheet1.ZIP › Original gels/Figure 3A/a┬-actin.tif]

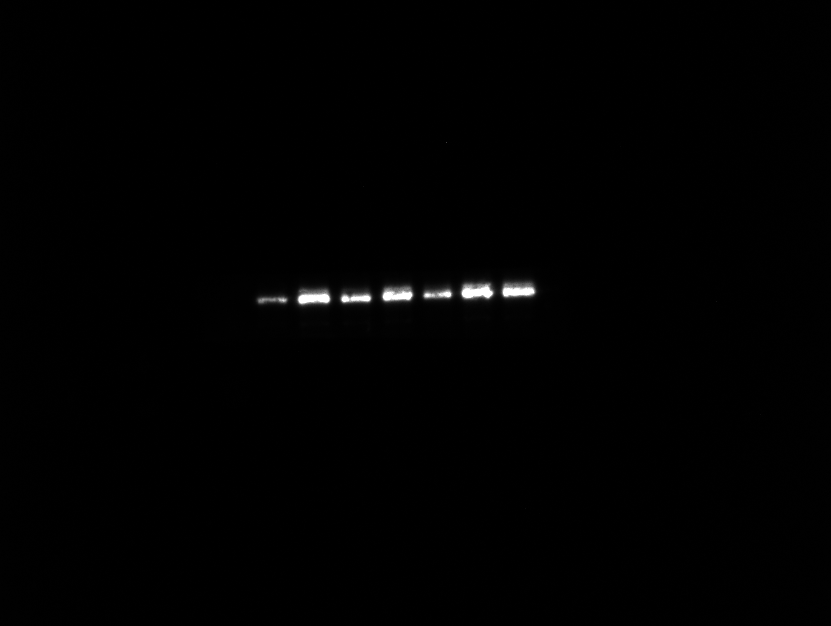

Supplement: Supplementary file 3 [file DataSheet1.ZIP › Original gels/Figure 3B/c-Jun.png]

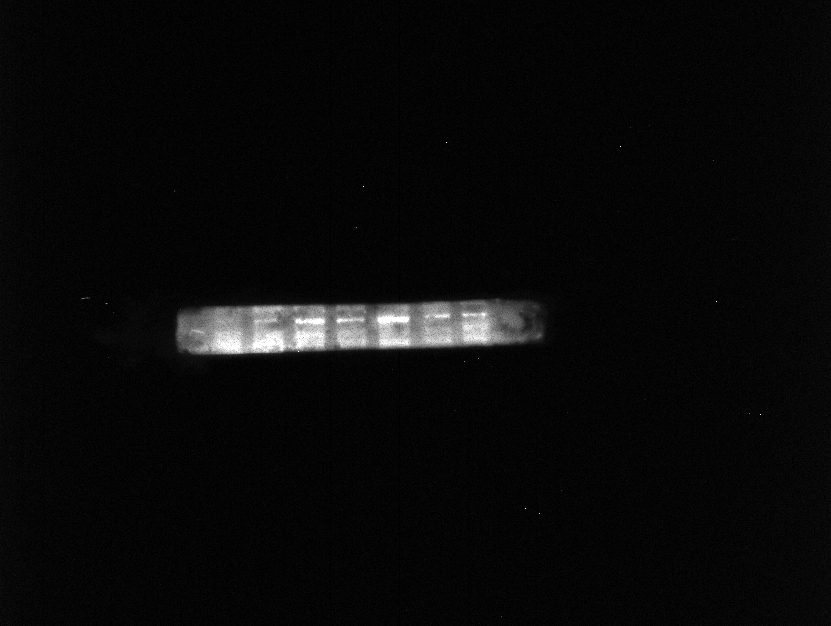

Supplement: Supplementary file 3 [file DataSheet1.ZIP › Original gels/Figure 3B/Nur77.png]

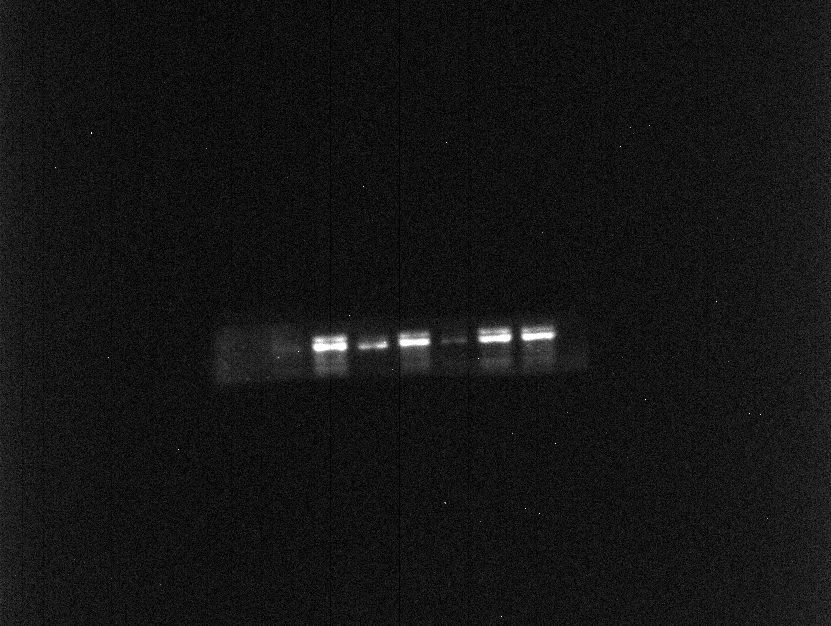

Supplement: Supplementary file 3 [file DataSheet1.ZIP › Original gels/Figure 3B/p-c-Jun.png]

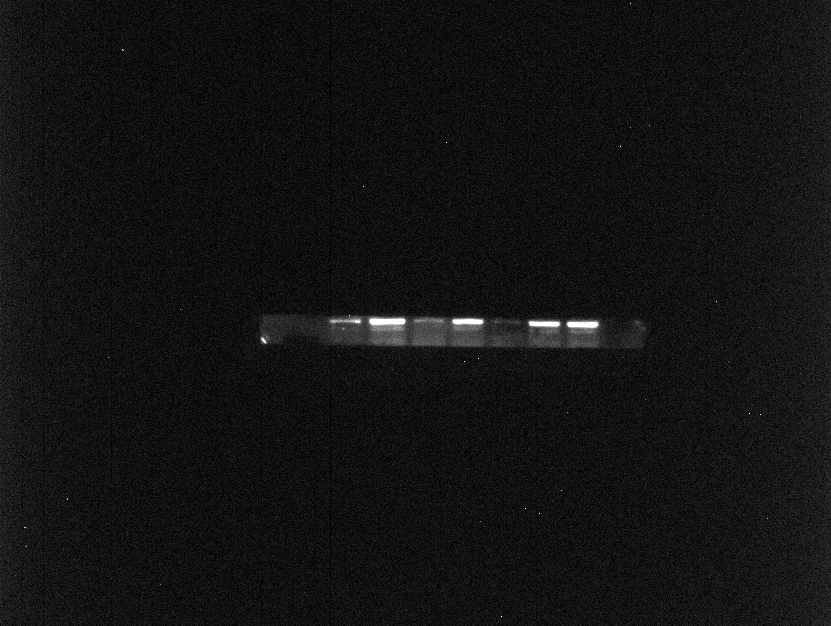

Supplement: Supplementary file 3 [file DataSheet1.ZIP › Original gels/Figure 3B/p-Nur77.png]

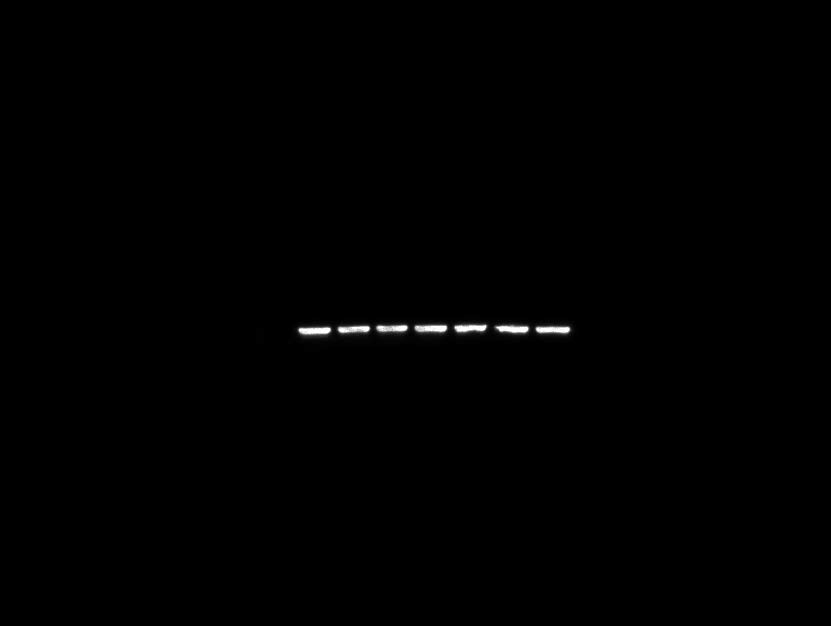

Supplement: Supplementary file 3 [file DataSheet1.ZIP › Original gels/Figure 3B/a┬-actin.png]

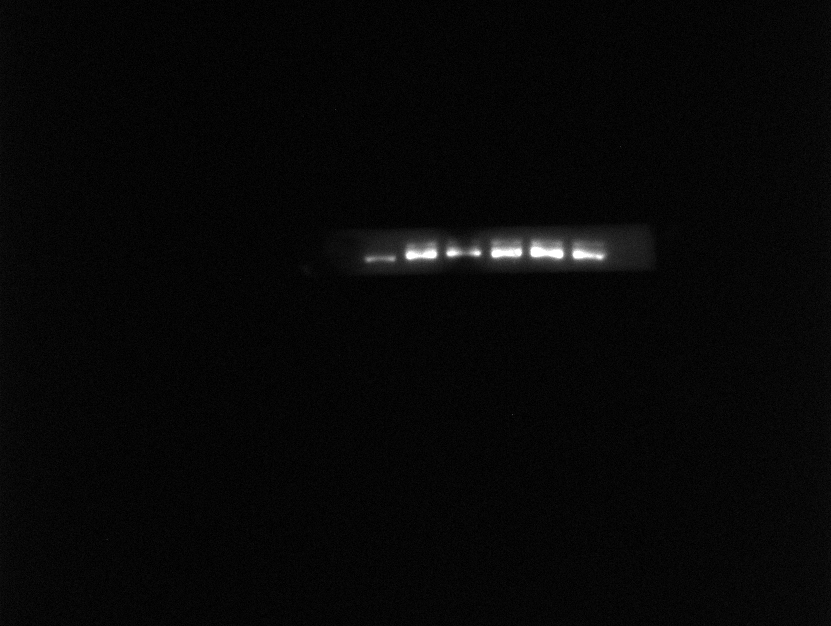

Supplement: Supplementary file 3 [file DataSheet1.ZIP › Original gels/Figure 4/c-Jun.png]

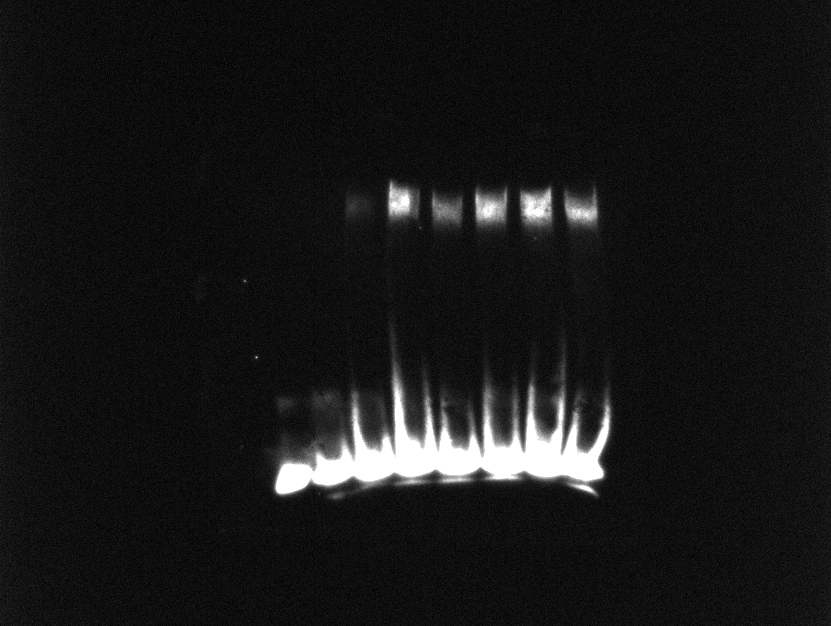

Supplement: Supplementary file 3 [file DataSheet1.ZIP › Original gels/Figure 4/EMSA.png]

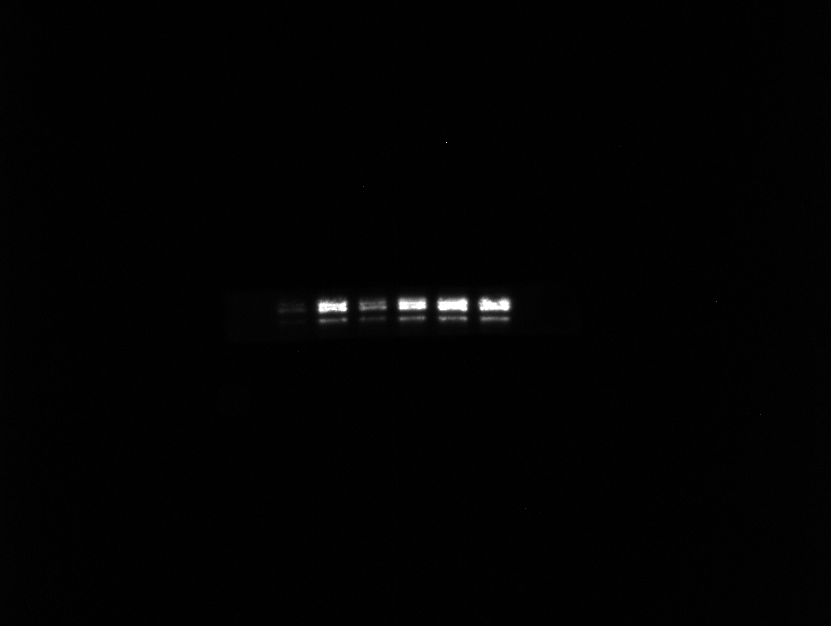

Supplement: Supplementary file 3 [file DataSheet1.ZIP › Original gels/Figure 4/p-c-Jun.png]

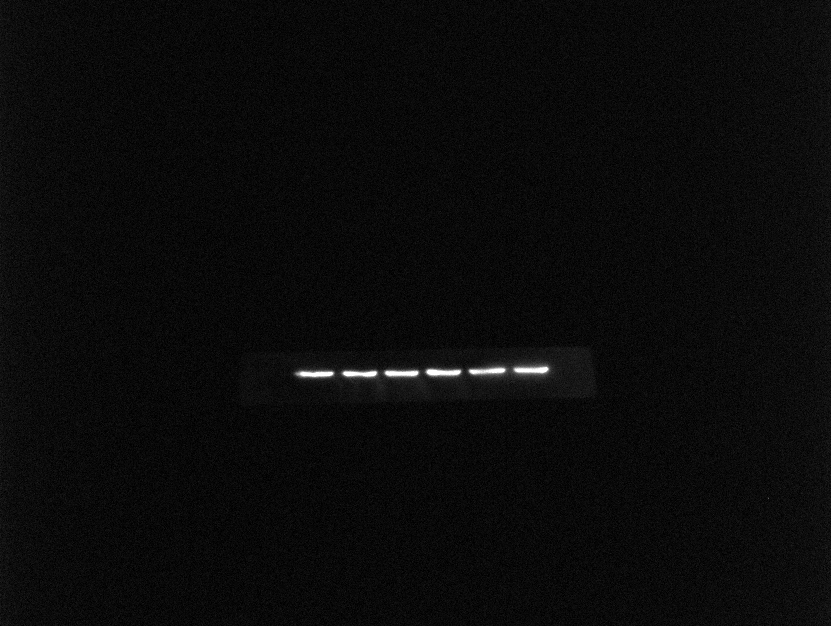

Supplement: Supplementary file 3 [file DataSheet1.ZIP › Original gels/Figure 4/a┬-actin.png]

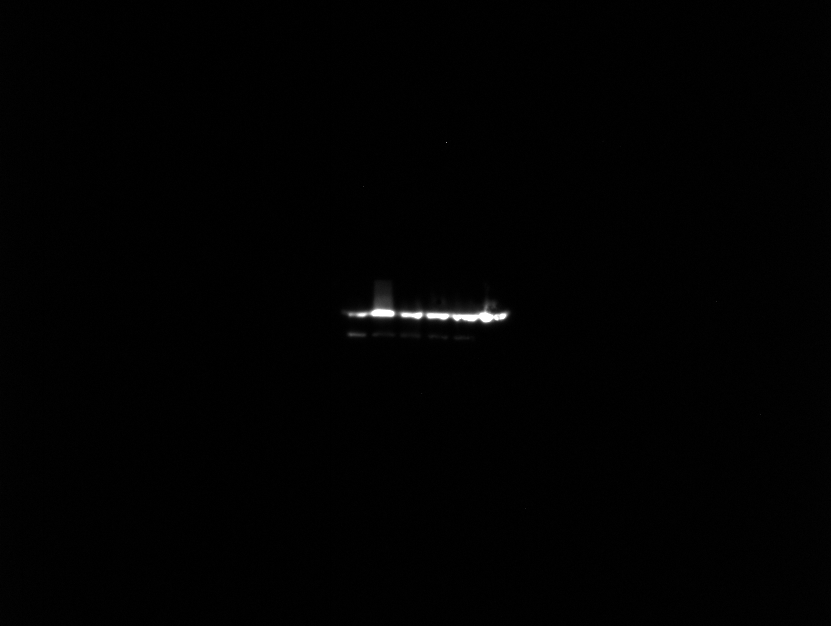

Supplement: Supplementary file 3 [file DataSheet1.ZIP › Original gels/Figure 6/c-Jun.png]

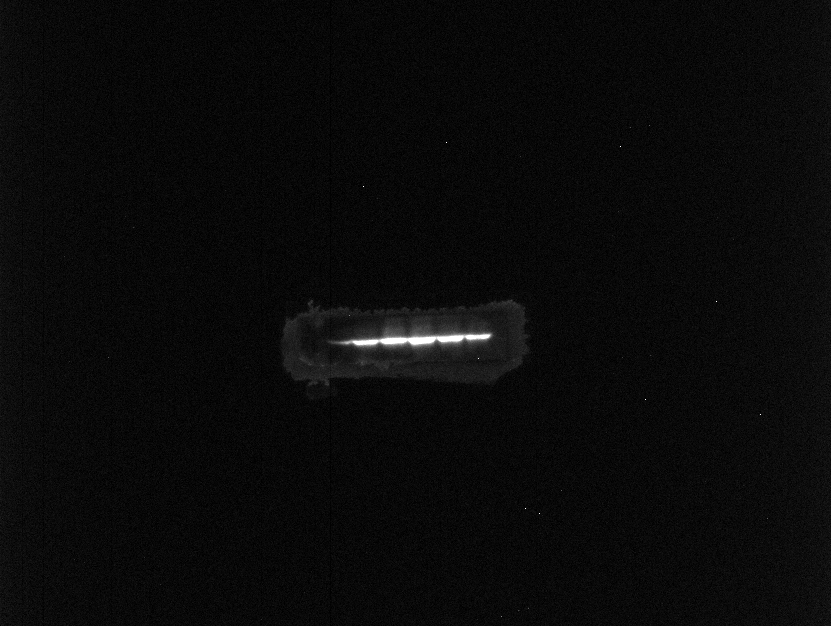

Supplement: Supplementary file 3 [file DataSheet1.ZIP › Original gels/Figure 6/Nur77.png]

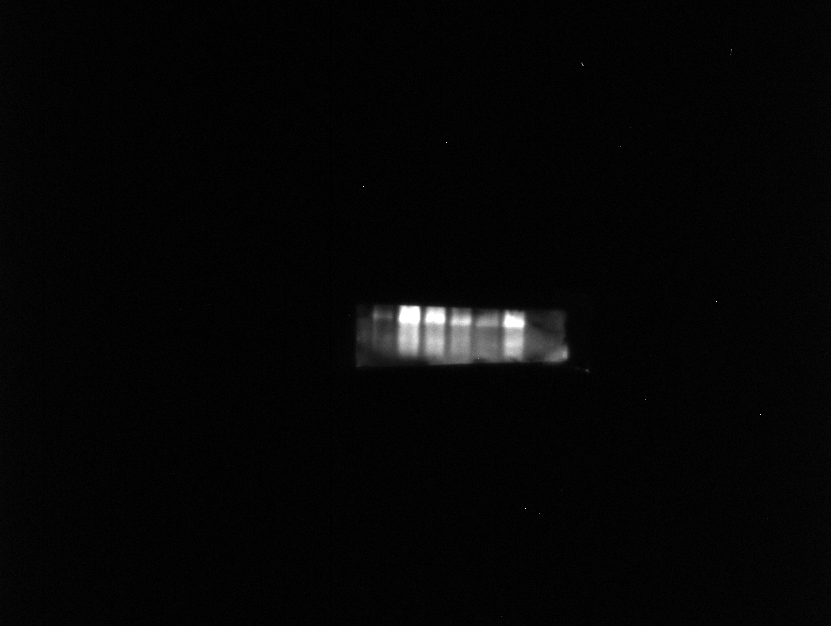

Supplement: Supplementary file 3 [file DataSheet1.ZIP › Original gels/Figure 6/p-c-Jun.png]

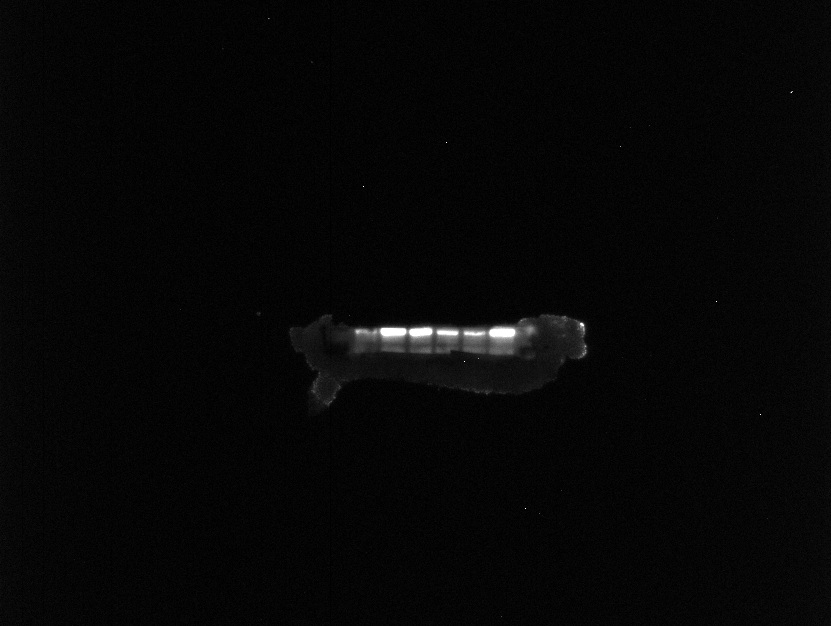

Supplement: Supplementary file 3 [file DataSheet1.ZIP › Original gels/Figure 6/p-Nur77.png]

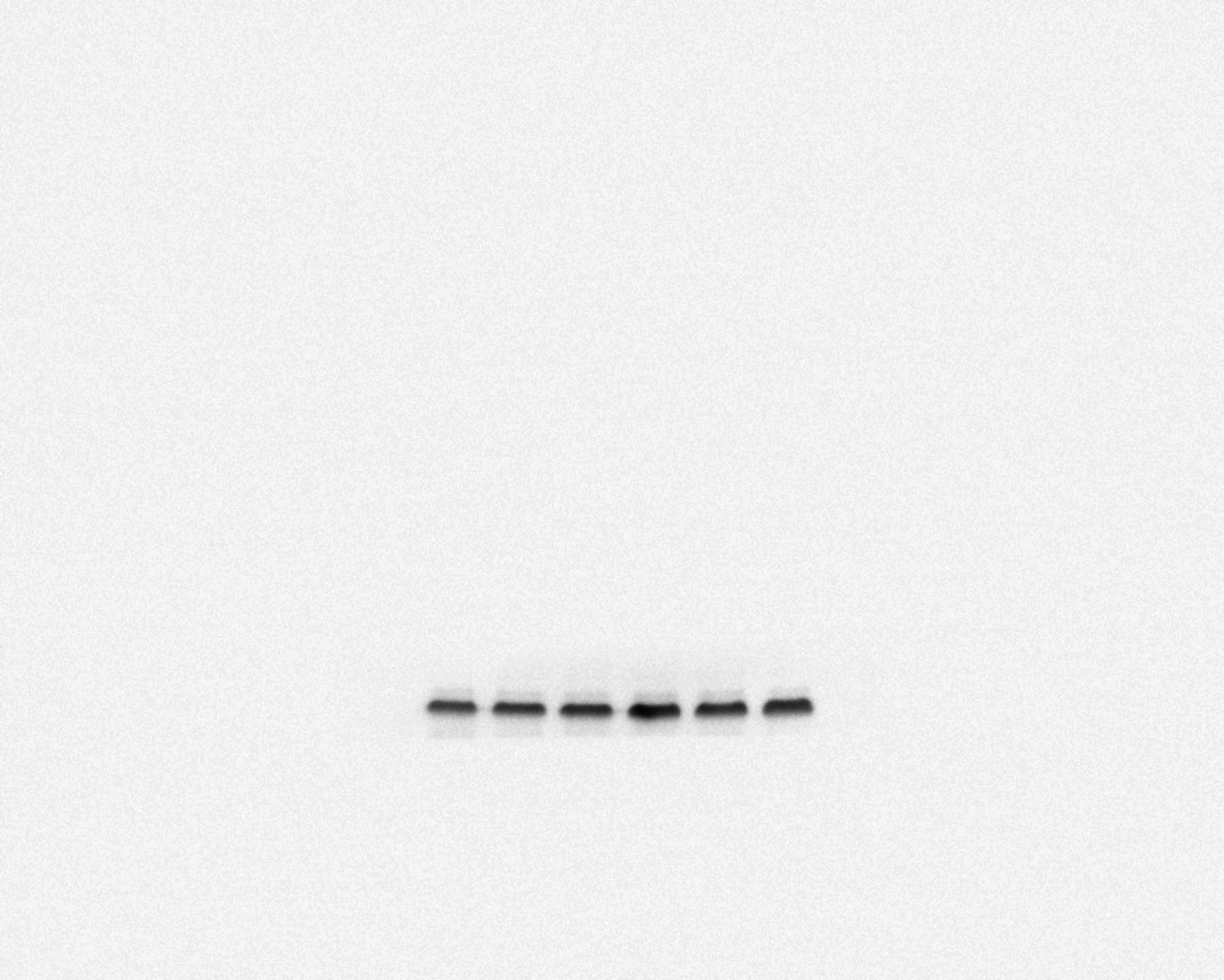

Supplement: Supplementary file 3 [file DataSheet1.ZIP › Original gels/Figure 6/a┬-actin.tif]

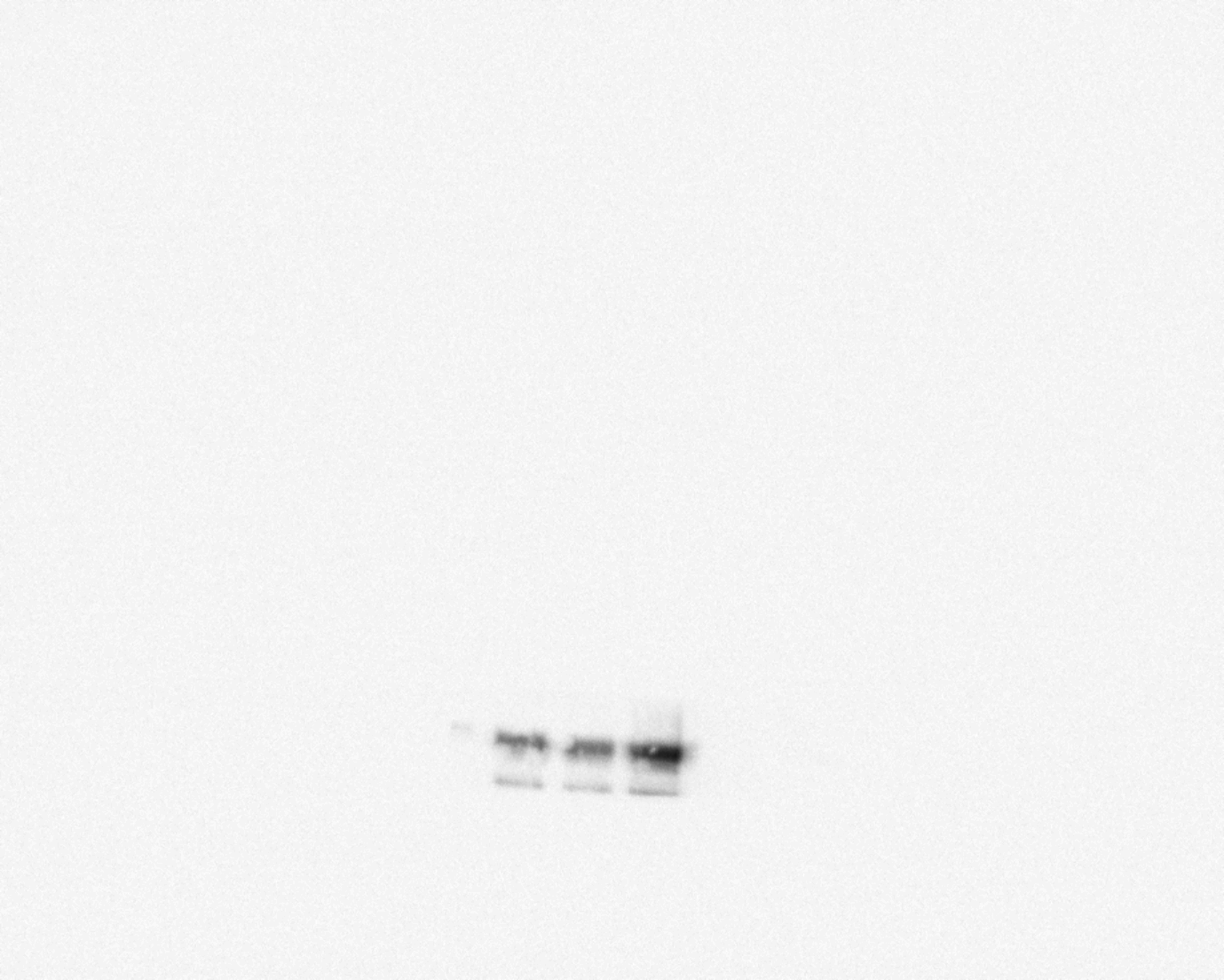

Supplement: Supplementary file 3 [file DataSheet1.ZIP › Original gels/Supplementary Figures/Supplementary Figure 3 JNK.tif]

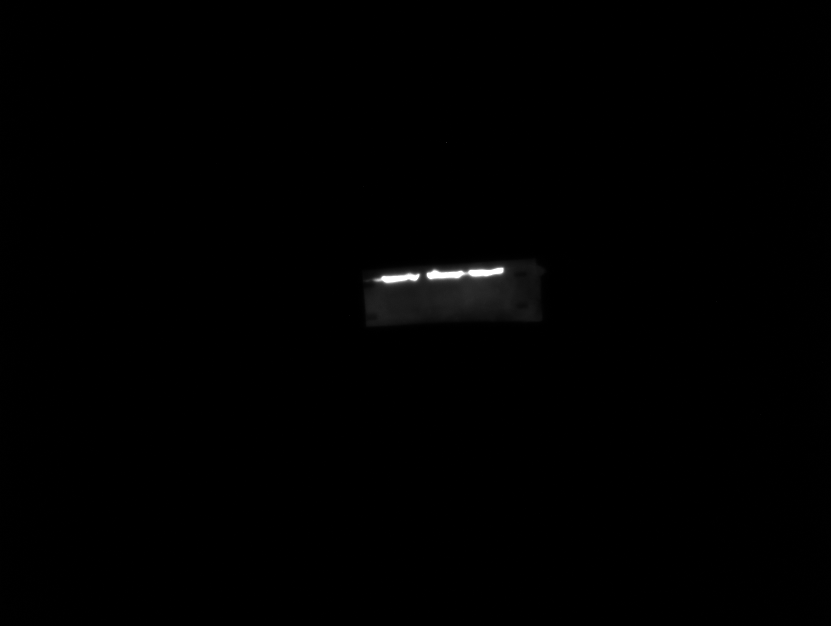

Supplement: Supplementary file 3 [file DataSheet1.ZIP › Original gels/Supplementary Figures/Supplementary Figure 3 a┬-actin.png]

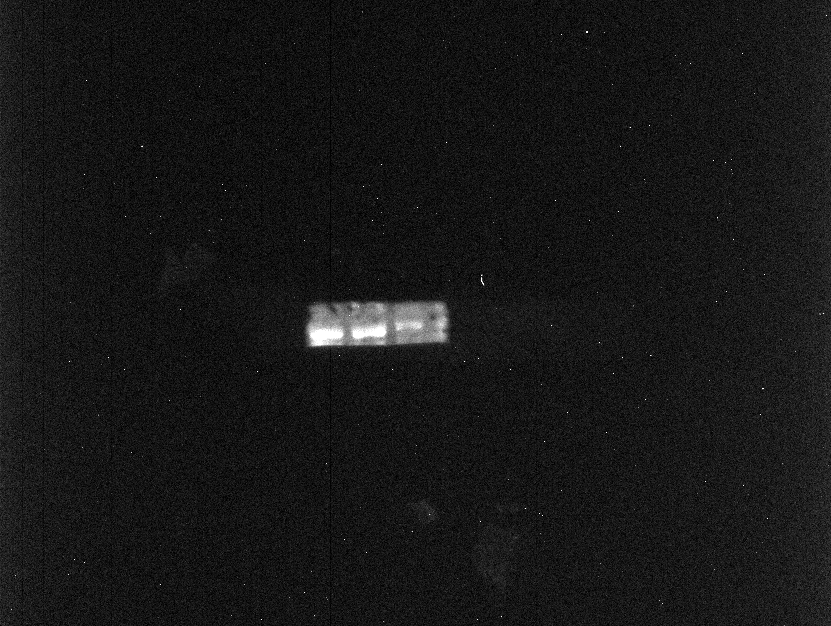

Supplement: Supplementary file 3 [file DataSheet1.ZIP › Original gels/Supplementary Figures/Supplementary Figure 4 Nur77.png]

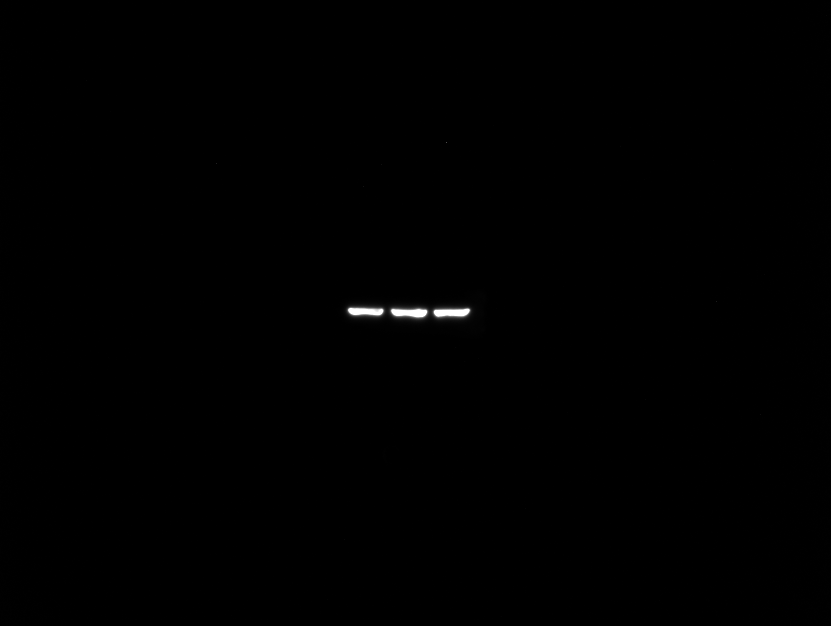

Supplement: Supplementary file 3 [file DataSheet1.ZIP › Original gels/Supplementary Figures/Supplementary Figure 4 a┬-actin.png]
